# Supplementary material for: The architecture of partisan debates: The online controversy on the no-deal Brexit
Source: PLoS One. 2022 Jun 30;17(6):e0270236. doi: 10.1371/journal.pone.0270236 (PMC9246126; doi:10.1371/journal.pone.0270236)
Supplement: S1 File — (PDF) [file pone.0270236.s001.pdf]

Supplementary Information

The architecture of partisan debates: The online controversy on the no-deal Brexit

Carlo R. M. A. Santagiustina*, Massimo Warglien

*Corresponding author, email: carlo.santagiustina@unive.it

Content summary

This PDF file includes:

- Supplementary text;
- Figs. S1 to S7;
- Tables S1 to S12;

In particular, Supplement Section S1 contains additional materials related to the results. It includes: the values of the parameters used for the final STM estimation (Table S1); counts (and shares) of extracted relations that have "no deal" in cause-side, by relation type and by faction (Table S2); a table containing counts (and shares) of tweets and retweets about the "no-deal" containing one or more causal verbs, by faction (Table S3); a table with counts (and shares) of collected Twitter posts about "no deal" by type and by faction (Table S4); a table with counts of extracted relations that have "no deal" as cause, by relation type, negation dummy and faction (Table S5); a summary table of top 10 tokens by topic and by faction (Table S6); a table containing estimated topic propensity covariate effects (Table S7); three figures representing respectively significant differences in topic proportions as a function of polar verb type (Figure S1), partisan faction (Figure S2), and negation (Figure S3) covariates.Robustness checks and description of parameters used in the STM are described in Section S2 of the Supplement. The section includes: the values of the parameters used in experiments for choosing the number of topics K (Table S8); two Figures representing the STM's *Lower bound* (Figure S6) and *Heldout likelihood* (Figure S5) as a function of K. Finally, details about the methods and code are given in Section S3, which contains: a summary of the workflow (Figure S7); the list of used stopwords (Table S3); a RegEx used to identify relations that mention the no-deal in the cause-side (Table S3); the list of verbs used to build verb phrases related to *Destruction*, *Causation* and *Creation* (Table S11); and the R code used to identify and extract cause-effect relations from tweets (Table S12).

S1 Supplementary Results

The results in the main paper and in this supplement have been obtained using the parameter values in Table S1 for estimating the STM. For not listed parameters, *Stm* (V.1.3.5) library defaults have been used. The model converged after 32 iterations. The value of the *Lower Bound* at the final iteration is −1203368.488309473032132.

**parameter**	**value**
K	38
Topic prevalence	$rel.type + neg + fct + s(t)$
Topic content	fct
max.em.its	500
emtol	$1e - 05$
init.type	Spectral
gamma.maxits	5000

Table S1: Parameter values used in the final STM estimation.

	Creation	Causation	Destruction	**TOT.**
Brexiteers	436 (49.7%)	325 (37.1%)	116 (13.2%)	877 (2.4%)
Others	15817 (46.0%)	13181 (38.3%)	5398 (15.7%)	34396 (95.2%)
Remainers	361 (42.8%)	334 (39.6%)	148 (17.6%)	843 (2.3%)
**TOT.**	16614 (46.0%)	13840 (38.3%)	5662 (15.7%)	36116

Pearson's Chi-squared test. (vars: *faction* and *rel. type*, data: *causal relations that "no deal" in cause-side*):
X-squared = 10.482, df = 4, p-value = 0.03305

Table S2: Counts (and shares) of extracted relations that have "no deal" in cause-side, by relation type and by faction, followed by Pearson's Chi-squared test.

	TWEETS with CM[1]	TWEETS without CM[1]
Brexiteers	7407 (11.9%)	54900 (88.1%)
Others	265879 (12.5%)	1853757 (87.5%)
Remainers	6304 (15.2%)	35161 (84.8%)

Pearson's Chi-squared test (vars: *faction* and *contains.causal.marker*, data: *tweets only*):
X-squared = 289.18, df = 2, p-value $< 10^{-15}$

	RETWEETS with CM[1]	RETWEETS without CM[1]
Brexiteers	28928 (14.5%)	170617 (85.5%)
Others	1024830 (16.0%)	5394586 (84.0%)
Remainers	27630 (17.0%)	134928 (83.0%)

Pearson's Chi-squared test (vars: *faction* and *contains.causal.marker*, data: *retweets only*):
X-squared = 448.21, df = 2, p-value $< 10^{-15}$

Table S3: Counts (and shares) of tweets and retweets about the "no-deal" containing one or more *Causal Markers* (CM), by faction, followed by Pearson's Chi-squared tests.

	TWEETS	RETWEETS	**FACTION TOT.**
Brexiteers	62307 (23.8%)	199545 (76.2%)	261852 (2.9%)
Others	2119636 (24.8%)	6419416 (75.2%)	8539052 (94.8%)
Remainers	41465 (20.3%)	162558 (79.7%)	204023 (2.3%)
**TYPE TOT.**	2223408 (24.7%)	6781519 (75.3%)	9004927

Pearson's Chi-squared test (vars: *faction* and *is.retweet*):
X-squared = 2285.7, df = 2, p-value $< 10^{-15}$

Table S4: Counts (and shares) of collected Twitter posts about "no deal" by type and by faction.

fct	N. (%)	*negated*	N. (%)	*rel.type*	N. (%)
Brexiteers	877 (2.4%)	FALSE	856 (97.6%)	Creation	423 (49.4%)
				Causation	319 (37.3%)
				Destruction	114 (13.3%)
		TRUE	21 (2.4%)	Creation	13 (61.9%)
				Causation	6 (28.6%)
				Destruction	2 (9.5%)
Others	34396 (95.2%)	FALSE	33343 (96.9%)	Creation	15162 (45.5%)
				Causation	12892 (38.7%)
				Destruction	5289 (15.8%)
		TRUE	1.053 (3.1%)	Creation	655 (62.2%)
				Causation	289 (27.4%)
				Destruction	109 (10.4%)
Remainers	843 (2.3%)	FALSE	813 (96.4%)	Creation	344 (42.3%)
				Causation	326 (40.1%)
				Destruction	143 (17.6%)
		TRUE	30 (3.6%)	Creation	17 (56.7%)
				Causation	8 (26.7%)
				Destruction	5 (16.6%)

Mantel-Haenszel chi-squared tests:
- strata: *fct*, vars: *rel.type* and *negated*, $M^2 = 118.81$, df = 2, p-value $< 10^{-14}$
- strata: *rel.type*, vars: *negated* and *fct*, $M^2 = 2.5141$, df = 2, p-value = 0.2845
- strata: *negated*, vars: *rel.type* and *fact*, $M^2 = 10.994$, df = 4, p-value= 0.02663

Table S5: Counts of extracted relations that have "no deal" as cause, by relation type, negation dummy and faction, followed by Mantel-Haenszel chi-squared tests.

Topic	Topic Prop.	Brexiteers (top 10 tokens)	Others (top 10 tokens)	Remainers (top 10 tokens)
24	4.9%	now, just, know, voting, see, happy, ever, well, fta, table	now, know, remain, sense, see, well, far, fact, last, remainers	far, now, know, right, anything, must, staff, year, saying, thinking
23	4.5%	no-deal, borisjohnson, illegal, says, likely, no-deal->brexit, pm, scotland, says->borisjohnson, may	no-deal, likely, borisjohnson, says, pm, says->borisjohnson, likely->says, no-deal->brexit, illegal, independence	no-deal, borisjohnson, likely, pm, says, outcome, likely->says, really, perhaps, says->borisjohnson
9	4.4%	unitedkingdom, businesses, leaving, imports, tariffs, competitive, 1, recession, outside, loss	unitedkingdom, recession, leaving, less, massive, businesses, crisis, tariffs, exports, unitedkingdom->economy	unitedkingdom, recession, leaving, massive, tariffs, living, £, unemployment, scotland->leaving, unitedkingdom->fishing
16	4.3%	europeanunion, law, wto, deal->europeanunion, end, clean, terms, europeanunion->law, 2017, trading	europeanunion, back, law, end, come, whole, terms, working, within, deal->europeanunion	europeanunion, law, unitedkingdom->europeanunion, nightmare, europeanunion->give, backstop, neighbours, sound, end, apply
20	4%	deal, get, leave, without, 🤣, good, mps, theresamay, 😂->🤣, difficult	deal, get, good, without, or, getting, negotiate, done, trying, good->deal	deal, remain, get, theresamay, best, revoke, good, done, put, travel
3	4%	brexit, likely, happen, less, finally, wrong, talk, +, 😏, patients	brexit, want, happen, really, possible, voters, less, +, idea, less->likely	brexit, peoplesvote, voted, brexit->europeanunion, wrong, constituents, vote->no-deal, failure, happen, finally
32	4%	economy, break, lives, world, harm, union, break->union, pound, rejoin, third	economy, damage, jobs, union, harm, lives, nhs, put, 10, risk	economy, jobs, damage, union, harm, break, nhs, huge, break->union, decades
28	3.9%	no, sense, difference, long, no->difference, actually, no->sense, vote, default, change	no, one, sense, difference, long, power, no->sense, change, no->difference, little	no, sense, uncertainty, change, difference, harder, long, longer, no->sense, position
33	3.8%	better, off, much, poorer, worse, better->off, threat, road, poorest, democratic	country, off, worse, much, poorer, things, difficult, worse->off, north, mean	less, poorer, off, leave, much, even, terms, lives, trade, democratic
11	3.4%	border, northernireland, months, chaos, hard, public, ireland, land, operation, northernireland->border	border, chaos, hard, public, hard->border, ireland, months, problem, minister, northernireland	chaos, public, months, border, disorder, hard, chaos->public, months->chaos, public->disorder, hard->border
26	3.4%	ireland, united->ireland, united, damage, government, labour, minimal, minimal->damage, labour->government, or	government, ireland, jeremycorbyn, take, northernireland, or, labour, damage, united, years	jeremycorbyn, northernireland, ireland, scotland, farming, mess, government, jeremycorbyn->government, loss->life, wales
35	2.9%	£, going, win, give, money, millions, power, exit, little, control	money, said, going, millions, tax, harder, give, win, pay, others	money, going, benefit, £, spending, financial, tax, fortune, buddies, hide
25	2.8%	people, want, still, believe, claiming, make, isnt, understand, thinks, die	people, still, believe, understand, national, million, die, emergency, customs, checks	people, want, still, believe, understand, lack, national, stopthecoup, make, die
18	2.8%	years, take, sun, or, trade, minimal, government, jeremycorbyn, labour, damage	years, trade, deals, new, world, another, civil, free, trade->deals, unrest	trade, years, civil, free, another, risk, unrest, friends, civil->unrest, nation

Table S6: Summary table of topics (by overall topic proportion), with top 10 tokens (by token probability) by topic and by faction. Row color scale represents significant (at the 0.01 significance level) differences of the estimated topic proportion coefficients of the two partisan factions. The closer is the row color to the corresponding faction color, the more characterizing is a specific topic for one of the two partisan faction. Characterizing topics for *Remainers* are in blue, whereas characterizing topics for *Brexiteers* are in red.

Table S6 – *Continued on next page*

Table S6 – *Continued from previous page*

Topic	Topic Prop.	Brexiteers (top 10 tokens)	Others (top 10 tokens)	Remainers (top 10 tokens)
21	2.8%	like, look, theresamay, look->like, left, warning, fishing, brexit->fishing, fishing->warning, sacrifice->brexit, theresamay->sacrifice	like, look, theresamay, 1, look->like, thing, shit, seem, big, best	like, look, look->like, seem->like, seem, poor, fault, thing, extremely, fool
34	2.7%	offer, voted, position, million, things, way, result, bad, 17.4, negotiation	better, way, bad, work, life, lose, option, anyone, everything, loss	better, life, worse, loss, anyone, situation, bad, plan, way, things
37	2.7%	already, many, real, term, sides, said, deaths, mass, proper, short	many, deaths, already, term, short, shortage, hardship, mass, short->term, pain	many, deaths, misery, meds, proper, avoidable->deaths, avoidable, real, short, officials->say
31	2.6%	😂, economic, hit, non, year, german, economic->problems, poor, problems, no-deal->default	economic, time, say, unitedkingdom, 😂, disaster, companies, actually, hit, recession	economic, crisis, impossible, companies, utter, disruption, social, economic->crisis, norway, german
15	2.4%	let, shortage, huge, thus, disruption, dogging, alone, water, let->alone, problems	problems, disruption, huge, 3, medicines, supply, delays, including, price, serious	disruption, medical, supplies, let, supply, cancer, drugs, medicines, increase, petrol
5	2.4%	50, article, article->50, party, labour, heard, no-deal, tory, default, mp	party, tory, election, labour, majority, general, must, tory->party, brexit->party, conservative	vote, party, labour, tory, tory->party, ok, changes, leader, place, general
17	2.4%	greatbritain, great, greatbritain->great, country, suffer, 2019, democracy, greatbritain->people, save, stupid	greatbritain, great, break, left, democracy, agree, farming, though, suffer, england	country, greatbritain, take, democracy, single, uklabour, much->damage, 2019, account, entire
7	2.2%	industry, business, stock, car->industry, products, global, car, entire, getting, experts	business, right, industry, greatbritain, thousands, state, manufacturing, car, services, orchids	manufacturing, industry, global, thousands, reputation, international, manufacturing->industry, investment, car, cars
10	2.2%	impossible, stop, revolution, peaceful, parliament, extension, ge, fucking, gov, main	parliament, stop, negotiations, position, impossible, ge, negotiating, least, expensive, taking	negotiations, stop, parliament, decade, expensive, lying, uncertainty, always, block, 100
36	2.2%	even, billions, tories, blame, half, bad->deal, even->though, though, super, even->if	even, tories, everyone, billions, rich, blame, care, richer, else, generation	even, tories, vulnerable, rich, already, though, blame, generation, billions, everyone
19	2.1%	fall, laws, prices, 30, rights, sky, market, high, lower, shops	£, year, prices, pound, billion, rights, fall, lower, 100, higher	problems, businesses, citizens, terrible, terrible->problems, problems->citizens, citizens->businesses, prices, lower, kids
2	2.1%	food, GB, shortages, riots, food->shortages, 👍, cost, government, held, apparently	food, shortages, job, medicine, losses, job->losses, collapse, food->shortages, medicine->shortages, riots	shortages, job, food, medicine, food->shortages, medication, GB, medicine->shortages, job->losses, losses

Table S6 – *Continued on next page*

Table S6 – *Continued from previous page*

Topic	Topic Prop.	Brexiteers (top 10 tokens)	Others (top 10 tokens)	Remainers (top 10 tokens)
1	1.9%	election, 🤮, general, next, majority, go, away, ge, next->election, next->ge	vote, go, referendum, away, decision, election, next, along, ge, campaign	go, ge, next, decision, away, suffering, campaign, next->ge, fully, go->away
30	1.9%	conservatives, point, 3, need, 10, unelectable, exactly, conservatives->need, exactly->conservatives, ago	voted, need, mps, 2, point, wto, first, place, something, rules	need, time, mean, point, lose->jobs, home, lots, fantasy, first, libdems
27	1.8%	new, two, germany, happened, europe, collapse, changing, really, becoming, france	scotland, europe, uncertainty, |, political, two, politics, sick, full, zero	or, getting, wants, sick, night, new, political, europe, existing, head
38	1.8%	general->election, or, not, just, europeanunion, deal, vote, no-deal, people, unitedkingdom	not, or, just, general->election, confirm, early->election, continued, future, keep, might	not, election, general->election, or, europeanunion, unitedkingdom, deal, people, just, no-deal
6	1.6%	if, one, happens, every, parties, art50, wonder, day, every->day, apart	if, every, day, mp, wales, wonder, avoid, happens, quickly, course	if, every, one, happens, apart, day, wonder, every->day, avoid, ministers
8	1.6%	leave, 31, leave->europeanunion, worst, benn, october, ready, case, mild, disruption	leave, case, worst, issue, show, foreign, office, pass, says, voted->leave	act, case, momentum, benn, benn->act, designed, boss, roi, worst, facts
12	1.6%	act, backstop, agreed, withdrawal, choice, agreement, situation, agree, withdrawal->act, ask	extension, backstop, agreement, choice, act, withdrawal, since, revoke, ask, drop	everything, 50, fall, career, choice, article, pound, agreement, limit, force
22	1.6%	outbreak, scurvy, outbreak->scurvy, wait, death, enough, available, streets, across, mailonline	50, article, article->50, death, across, outbreak, scurvy, outbreak->scurvy, wait, streets	prime, prime->minister, minister, available, easily, across->country, agree, streets, across, cameron
13	1.5%	sure, remainers, date, leavers, maybe, almost, come, certain, massive, hopefully	sure, never, ever, enough, almost, leavers, gets, 🤔, march, certain	default, government, revokea50, option, referendum, legal, unitedkingdom->government, come, sure, revokea50->default
4	1.1%	live, blocking->roads, farmers->blocking, blocking, roads->protest, protest, farmers, roads, news, move	news, next, live, move, farmers, live->news, 4, stage, blocking, next->stage	business, stage, lost, news, live, next, roads, move->next, next->stage, stage->live
14	1.1%	clear, referendum, accept, nothing, name, behind, ridiculous, second, made, perfectly->clear	clear, nothing, second, accept, major, ref, second->referendum, hands, behind, indy	clear, nothing, 2nd, fact, accept, table, ref, behind, second, ridiculous
29	0.9%	not, time, losing, claims, pointless, votes, or->not, society, something, commons	default, disaster, legal, votes, greater, damage->economy, position, whether, society, legislation	just, not->just, society, revocation, greater, not, less->competitive, competitive, economically, power

Table S7: Estimated topic propensity parameters

topic	term	estimate	std.error	statistic	p.value
1	Constant	0.0160	0.0023	7.0273	0.0000
1	*rel.type*=Creation	0.0083	0.0008	10.8517	0.0000
1	*rel.type*=Destruction	0.0078	0.0011	7.0078	0.0000
1	*neg*=TRUE	0.0165	0.0025	6.4734	0.0000
1	*fct*=Other	0.0006	0.0023	0.2592	0.7955
1	*fct*=Remainer	0.0012	0.0034	0.3577	0.7206

Continued on next page

Table S7 – *Continued from previous page*

topic	term	estimate	std.error	statistic	p.value
2	Constant	0.0405	0.0032	12.5478	0.0000
2	*rel.type*=Creation	-0.0394	0.0013	-31.0073	0.0000
2	*rel.type*=Destruction	-0.0387	0.0016	-24.5399	0.0000
2	*neg*=TRUE	-0.0038	0.0028	-1.3641	0.1725
2	*fct*=Other	0.0088	0.0029	2.9790	0.0029
2	*fct*=Remainer	0.0062	0.0045	1.3652	0.1722
3	Constant	0.0221	0.0023	9.7882	0.0000
3	*rel.type*=Creation	0.0100	0.0008	12.4265	0.0000
3	*rel.type*=Destruction	0.0244	0.0011	22.7367	0.0000
3	*neg*=TRUE	0.0042	0.0021	2.0082	0.0446
3	*fct*=Other	0.0020	0.0023	0.8617	0.3889
3	*fct*=Remainer	-0.0054	0.0032	-1.7141	0.0865
4	Constant	0.0157	0.0028	5.6770	0.0000
4	*rel.type*=Creation	0.0016	0.0009	1.7799	0.0751
4	*rel.type*=Destruction	-0.0019	0.0013	-1.4109	0.1583
4	*neg*=TRUE	-0.0073	0.0021	-3.4211	0.0006
4	*fct*=Other	0.0020	0.0029	0.6942	0.4875
4	*fct*=Remainer	-0.0028	0.0038	-0.7466	0.4553
5	Constant	0.0640	0.0040	15.8701	0.0000
5	*rel.type*=Creation	-0.0036	0.0011	-3.3020	0.0010
5	*rel.type*=Destruction	0.0297	0.0016	18.6011	0.0000
5	*neg*=TRUE	-0.0026	0.0023	-1.1385	0.2549
5	*fct*=Other	-0.0405	0.0040	-10.0968	0.0000
5	*fct*=Remainer	-0.0416	0.0049	-8.5345	0.0000
6	Constant	0.0193	0.0020	9.5565	0.0000
6	*rel.type*=Creation	0.0033	0.0006	5.1408	0.0000
6	*rel.type*=Destruction	0.0042	0.0009	4.6849	0.0000
6	*neg*=TRUE	-0.0039	0.0016	-2.4214	0.0155
6	*fct*=Other	-0.0028	0.0020	-1.4046	0.1601
6	*fct*=Remainer	-0.0034	0.0031	-1.1154	0.2647
7	Constant	0.0106	0.0025	4.2538	0.0000
7	*rel.type*=Creation	-0.0005	0.0009	-0.6007	0.5480
7	*rel.type*=Destruction	0.0421	0.0014	29.5135	0.0000
7	*neg*=TRUE	-0.0081	0.0022	-3.6805	0.0002
7	*fct*=Other	0.0085	0.0025	3.4044	0.0007
7	*fct*=Remainer	0.0057	0.0037	1.5426	0.1229
8	Constant	0.0246	0.0029	8.4942	0.0000
8	*rel.type*=Creation	0.0046	0.0009	5.2425	0.0000

Continued on next page

topic	term	estimate	std.error	statistic	p.value
8	*rel.type*=Destruction	-0.0051	0.0010	-4.9672	0.0000
8	*neg*=TRUE	0.0008	0.0021	0.3784	0.7051
8	*fct*=Other	-0.0059	0.0029	-2.0451	0.0408
8	*fct*=Remainer	-0.0146	0.0036	-4.0617	0.0000
9	Constant	0.0388	0.0027	14.2883	0.0000
9	*rel.type*=Creation	-0.0175	0.0009	-18.9675	0.0000
9	*rel.type*=Destruction	-0.0034	0.0012	-2.8020	0.0051
9	*neg*=TRUE	-0.0043	0.0023	-1.8385	0.0660
9	*fct*=Other	0.0063	0.0028	2.2785	0.0227
9	*fct*=Remainer	0.0091	0.0040	2.2853	0.0223
10	Constant	0.0252	0.0026	9.6413	0.0000
10	*rel.type*=Creation	0.0080	0.0007	10.8194	0.0000
10	*rel.type*=Destruction	0.0006	0.0010	0.5435	0.5868
10	*neg*=TRUE	0.0018	0.0023	0.7796	0.4356
10	*fct*=Other	-0.0057	0.0026	-2.2104	0.0271
10	*fct*=Remainer	-0.0150	0.0034	-4.4076	0.0000
11	Constant	0.0398	0.0036	11.1730	0.0000
11	*rel.type*=Creation	-0.0275	0.0013	-20.4315	0.0000
11	*rel.type*=Destruction	-0.0334	0.0016	-20.6136	0.0000
11	*neg*=TRUE	0.0139	0.0039	3.5857	0.0003
11	*fct*=Other	0.0149	0.0035	4.2055	0.0000
11	*fct*=Remainer	0.0237	0.0051	4.6690	0.0000
12	Constant	0.0228	0.0023	9.9259	0.0000
12	*rel.type*=Creation	-0.0010	0.0008	-1.3111	0.1898
12	*rel.type*=Destruction	-0.0071	0.0011	-6.2938	0.0000
12	*neg*=TRUE	0.0121	0.0027	4.5758	0.0000
12	*fct*=Other	-0.0018	0.0023	-0.7822	0.4341
12	*fct*=Remainer	0.0016	0.0036	0.4491	0.6534
13	Constant	0.0132	0.0020	6.6517	0.0000
13	*rel.type*=Creation	0.0144	0.0007	21.3358	0.0000
13	*rel.type*=Destruction	0.0021	0.0008	2.6756	0.0075
13	*neg*=TRUE	-0.0044	0.0018	-2.4675	0.0136
13	*fct*=Other	-0.0031	0.0019	-1.6165	0.1060
13	*fct*=Remainer	0.0213	0.0030	7.0397	0.0000
14	Constant	0.0148	0.0021	7.0230	0.0000
14	*rel.type*=Creation	0.0073	0.0007	10.6451	0.0000
14	*rel.type*=Destruction	-0.0007	0.0008	-0.8895	0.3738
14	*neg*=TRUE	-0.0042	0.0016	-2.6802	0.0074

Continued on next page

Table S7 – *Continued from previous page*

topic	term	estimate	std.error	statistic	p.value
14	*fct*=Other	-0.0030	0.0020	-1.5128	0.1303
14	*fct*=Remainer	-0.0004	0.0031	-0.1306	0.8961
15	Constant	0.0393	0.0025	15.5097	0.0000
15	*rel.type*=Creation	-0.0235	0.0009	-26.9684	0.0000
15	*rel.type*=Destruction	-0.0225	0.0012	-18.8189	0.0000
15	*neg*=TRUE	0.0122	0.0024	5.1409	0.0000
15	*fct*=Other	0.0001	0.0025	0.0584	0.9534
15	*fct*=Remainer	-0.0067	0.0035	-1.9169	0.0553
16	Constant	0.0547	0.0031	17.4128	0.0000
16	*rel.type*=Creation	0.0092	0.0008	11.6683	0.0000
16	*rel.type*=Destruction	0.0081	0.0012	6.9380	0.0000
16	*neg*=TRUE	-0.0026	0.0022	-1.1575	0.2471
16	*fct*=Other	-0.0267	0.0030	-8.8965	0.0000
16	*fct*=Remainer	-0.0357	0.0039	-9.1678	0.0000
17	Constant	0.0287	0.0026	11.2354	0.0000
17	*rel.type*=Creation	0.0081	0.0008	10.7616	0.0000
17	*rel.type*=Destruction	0.0178	0.0011	16.1294	0.0000
17	*neg*=TRUE	-0.0061	0.0018	-3.4714	0.0005
17	*fct*=Other	-0.0114	0.0026	-4.3874	0.0000
17	*fct*=Remainer	-0.0095	0.0033	-2.8660	0.0042
18	Constant	0.0396	0.0035	11.4103	0.0000
18	*rel.type*=Creation	-0.0032	0.0011	-2.9508	0.0032
18	*rel.type*=Destruction	0.0003	0.0014	0.2240	0.8228
18	*neg*=TRUE	0.0101	0.0027	3.7413	0.0002
18	*fct*=Other	-0.0083	0.0035	-2.3435	0.0191
18	*fct*=Remainer	-0.0111	0.0043	-2.5963	0.0094
19	Constant	0.0285	0.0034	8.3680	0.0000
19	*rel.type*=Creation	-0.0088	0.0011	-7.9786	0.0000
19	*rel.type*=Destruction	-0.0102	0.0014	-7.2765	0.0000
19	*neg*=TRUE	-0.0085	0.0026	-3.3223	0.0009
19	*fct*=Other	0.0035	0.0033	1.0435	0.2967
19	*fct*=Remainer	0.0108	0.0043	2.5015	0.0124
20	Constant	0.0412	0.0032	12.9566	0.0000
20	*rel.type*=Creation	0.0220	0.0010	22.1303	0.0000
20	*rel.type*=Destruction	0.0026	0.0012	2.1172	0.0342
20	*neg*=TRUE	0.0233	0.0033	7.0589	0.0000
20	*fct*=Other	-0.0167	0.0032	-5.1865	0.0000
20	*fct*=Remainer	-0.0160	0.0038	-4.2169	0.0000

Continued on next page

Table S7 – *Continued from previous page*

topic	term	estimate	std.error	statistic	p.value
21	Constant	0.0171	0.0026	6.4564	0.0000
21	*rel.type*=Creation	0.0207	0.0010	21.0634	0.0000
21	*rel.type*=Destruction	0.0092	0.0012	7.8525	0.0000
21	*neg*=TRUE	-0.0117	0.0023	-5.1587	0.0000
21	*fct*=Other	0.0002	0.0026	0.0704	0.9438
21	*fct*=Remainer	0.0006	0.0037	0.1568	0.8754
22	Constant	0.0288	0.0029	9.9498	0.0000
22	*rel.type*=Creation	-0.0247	0.0011	-23.0179	0.0000
22	*rel.type*=Destruction	-0.0200	0.0014	-14.4377	0.0000
22	*neg*=TRUE	-0.0050	0.0024	-2.0774	0.0378
22	*fct*=Other	0.0071	0.0029	2.4084	0.0160
22	*fct*=Remainer	-0.0061	0.0038	-1.6008	0.1094
23	Constant	0.0238	0.0034	7.0785	0.0000
23	*rel.type*=Creation	0.0298	0.0012	25.9190	0.0000
23	*rel.type*=Destruction	0.0000	0.0014	0.0041	0.9967
23	*neg*=TRUE	-0.0119	0.0028	-4.3161	0.0000
23	*fct*=Other	0.0022	0.0034	0.6454	0.5186
23	*fct*=Remainer	0.0017	0.0046	0.3641	0.7158
24	Constant	0.0354	0.0029	12.3901	0.0000
24	*rel.type*=Creation	0.0157	0.0008	20.1294	0.0000
24	*rel.type*=Destruction	0.0047	0.0011	4.3537	0.0000
24	*neg*=TRUE	0.0195	0.0026	7.4187	0.0000
24	*fct*=Other	-0.0065	0.0028	-2.3026	0.0213
24	*fct*=Remainer	-0.0074	0.0035	-2.1327	0.0330
25	Constant	0.0221	0.0023	9.5598	0.0000
25	*rel.type*=Creation	-0.0010	0.0007	-1.4067	0.1595
25	*rel.type*=Destruction	0.0142	0.0012	12.1276	0.0000
25	*neg*=TRUE	-0.0070	0.0019	-3.6501	0.0003
25	*fct*=Other	0.0030	0.0023	1.3099	0.1902
25	*fct*=Remainer	0.0059	0.0031	1.9232	0.0545
26	Constant	0.0395	0.0032	12.1924	0.0000
26	*rel.type*=Creation	-0.0303	0.0012	-24.6338	0.0000
26	*rel.type*=Destruction	-0.0192	0.0017	-11.1510	0.0000
26	*neg*=TRUE	-0.0037	0.0030	-1.2225	0.2215
26	*fct*=Other	0.0131	0.0032	4.1555	0.0000
26	*fct*=Remainer	0.0066	0.0043	1.5310	0.1258
27	Constant	0.0171	0.0024	7.0375	0.0000
27	*rel.type*=Creation	-0.0028	0.0009	-3.2232	0.0013

Continued on next page

Table S7 – *Continued from previous page*

topic	term	estimate	std.error	statistic	p.value
27	*rel.type*=Destruction	-0.0076	0.0011	-6.6079	0.0000
27	*neg*=TRUE	-0.0040	0.0021	-1.9116	0.0559
27	*fct*=Other	0.0073	0.0024	3.0478	0.0023
27	*fct*=Remainer	0.0066	0.0034	1.9263	0.0541
28	Constant	0.0284	0.0033	8.5978	0.0000
28	*rel.type*=Creation	0.0228	0.0011	21.6190	0.0000
28	*rel.type*=Destruction	-0.0040	0.0013	-3.1698	0.0015
28	*neg*=TRUE	0.0187	0.0036	5.1774	0.0000
28	*fct*=Other	-0.0030	0.0034	-0.8910	0.3729
28	*fct*=Remainer	0.0014	0.0043	0.3351	0.7376
29	Constant	0.0211	0.0023	9.3169	0.0000
29	*rel.type*=Creation	0.0030	0.0006	5.1266	0.0000
29	*rel.type*=Destruction	-0.0030	0.0008	-3.7888	0.0002
29	*neg*=TRUE	-0.0013	0.0017	-0.7728	0.4397
29	*fct*=Other	-0.0087	0.0022	-3.8932	0.0001
29	*fct*=Remainer	-0.0016	0.0029	-0.5623	0.5739
30	Constant	0.0132	0.0019	6.9358	0.0000
30	*rel.type*=Creation	0.0031	0.0007	4.1969	0.0000
30	*rel.type*=Destruction	-0.0005	0.0010	-0.5347	0.5929
30	*neg*=TRUE	-0.0034	0.0018	-1.8495	0.0644
30	*fct*=Other	0.0064	0.0018	3.4516	0.0006
30	*fct*=Remainer	-0.0041	0.0025	-1.5969	0.1103
31	Constant	0.0367	0.0029	12.5020	0.0000
31	*rel.type*=Creation	-0.0219	0.0011	-20.8501	0.0000
31	*rel.type*=Destruction	-0.0253	0.0013	-19.0093	0.0000
31	*neg*=TRUE	-0.0013	0.0030	-0.4191	0.6752
31	*fct*=Other	0.0063	0.0029	2.1692	0.0301
31	*fct*=Remainer	-0.0018	0.0041	-0.4389	0.6607
32	Constant	0.0315	0.0026	12.2313	0.0000
32	*rel.type*=Creation	-0.0247	0.0009	-26.9047	0.0000
32	*rel.type*=Destruction	0.0189	0.0014	13.8506	0.0000
32	*neg*=TRUE	-0.0053	0.0025	-2.1541	0.0312
32	*fct*=Other	0.0113	0.0026	4.4257	0.0000
32	*fct*=Remainer	0.0278	0.0046	6.0781	0.0000
33	Constant	0.0075	0.0025	2.9559	0.0031
33	*rel.type*=Creation	0.0230	0.0009	26.6673	0.0000
33	*rel.type*=Destruction	0.0296	0.0013	23.6445	0.0000
33	*neg*=TRUE	-0.0117	0.0021	-5.4961	0.0000

Continued on next page

Table S7 – *Continued from previous page*

topic	term	estimate	std.error	statistic	p.value
33	*fct*=Other	0.0108	0.0025	4.3929	0.0000
33	*fct*=Remainer	0.0213	0.0038	5.6308	0.0000
34	Constant	0.0232	0.0022	10.5312	0.0000
34	*rel.type*=Creation	0.0100	0.0007	15.1427	0.0000
34	*rel.type*=Destruction	0.0009	0.0010	0.9221	0.3565
34	*neg*=TRUE	0.0021	0.0021	1.0075	0.3137
34	*fct*=Other	-0.0034	0.0022	-1.5807	0.1140
34	*fct*=Remainer	-0.0021	0.0029	-0.7178	0.4729
35	Constant	0.0109	0.0021	5.2107	0.0000
35	*rel.type*=Creation	0.0189	0.0008	24.1147	0.0000
35	*rel.type*=Destruction	0.0021	0.0009	2.1888	0.0286
35	*neg*=TRUE	-0.0056	0.0020	-2.7687	0.0056
35	*fct*=Other	0.0074	0.0021	3.4534	0.0006
35	*fct*=Remainer	0.0009	0.0031	0.2990	0.7649
36	Constant	0.0069	0.0021	3.2615	0.0011
36	*rel.type*=Creation	0.0127	0.0008	15.9608	0.0000
36	*rel.type*=Destruction	0.0062	0.0011	5.8633	0.0000
36	*neg*=TRUE	-0.0073	0.0019	-3.8505	0.0001
36	*fct*=Other	0.0098	0.0021	4.7018	0.0000
36	*fct*=Remainer	0.0179	0.0033	5.4723	0.0000
37	Constant	0.0302	0.0023	13.1362	0.0000
37	*rel.type*=Creation	-0.0262	0.0009	-30.1670	0.0000
37	*rel.type*=Destruction	-0.0225	0.0012	-18.9386	0.0000
37	*neg*=TRUE	-0.0001	0.0023	-0.0411	0.9672
37	*fct*=Other	0.0118	0.0023	5.0894	0.0000
37	*fct*=Remainer	0.0131	0.0035	3.7157	0.0002
38	Constant	0.0074	0.0004	16.5572	0.0000
38	*rel.type*=Creation	0.0002	0.0002	1.0797	0.2803
38	*rel.type*=Destruction	-0.0003	0.0002	-1.2309	0.2184
38	*neg*=TRUE	-0.0001	0.0004	-0.3539	0.7234
38	*fct*=Other	0.0039	0.0005	8.6132	0.0000
38	*fct*=Remainer	0.0016	0.0006	2.5763	0.0100

Table S7: Estimated topic propensity parameters.

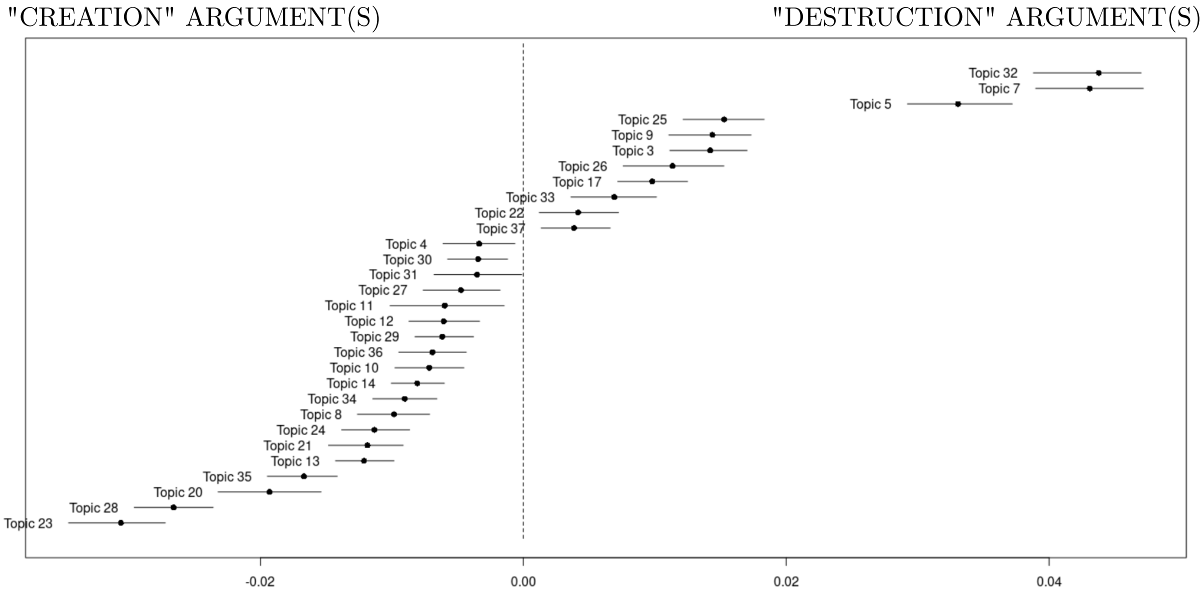

Figure S1: Significant estimated differences in argument k proportions (with 99% C.I.) of as a function of causal relation type rel: $\hat{\beta}_{k,rel=Destruction} - \hat{\beta}_{k,rel=Creation}$.

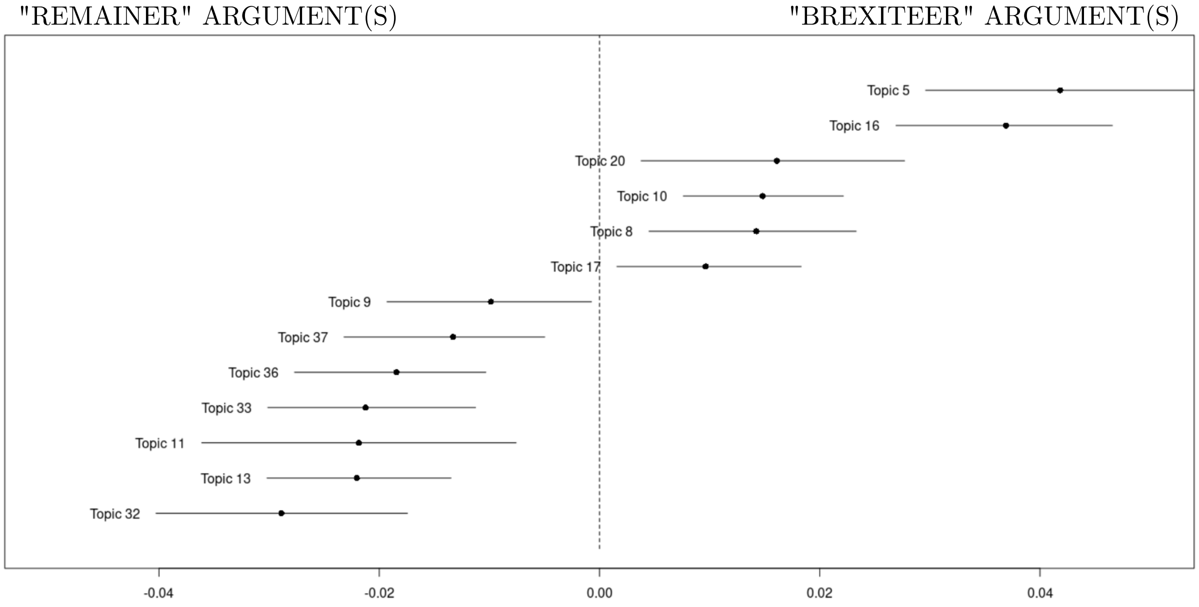

Figure S2: Significant estimated differences in topic k proportions (with 99% C.I.) of as a function of partisan faction fct: $\hat{\beta}_{k,fct=Brexiteer} - \hat{\beta}_{k,fct=Remaniner}$.

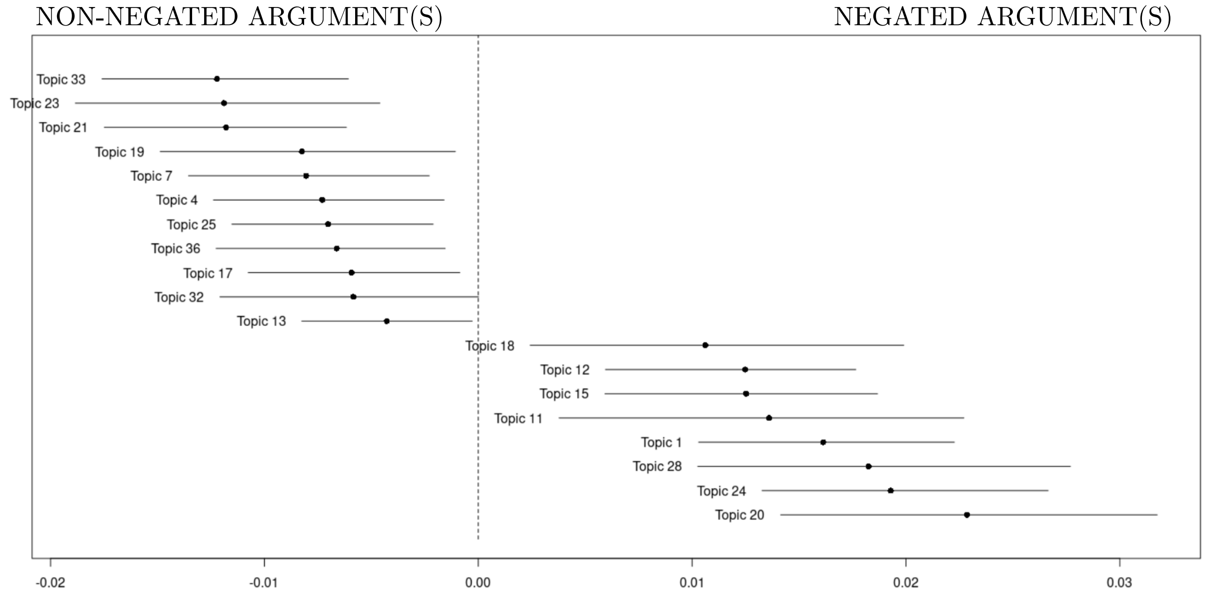

Figure S3: Significant estimated differences in topic k proportions (with 99% C.I.) of as a function of relation verb negation neg: $\hat{\beta}_{k,neg=TRUE} - \hat{\beta}_{k,neg=FALSE}$.

S2 Robustness Checks

**parameter**	**value**
K	c(3:70)
Number of experiment replications	50
Topic prevalence	$rel.type + neg + fct + s(t)$
Topic content	fct
Maximum Expectation Maximization Iterations	200
Expectations Maximization Tolerance	1e-05
Initialization Type	Spectral
Proportion of docs to be held out	25%
M	10

Table S8: Parameters used in experiments with different values for K from 3 to 70.

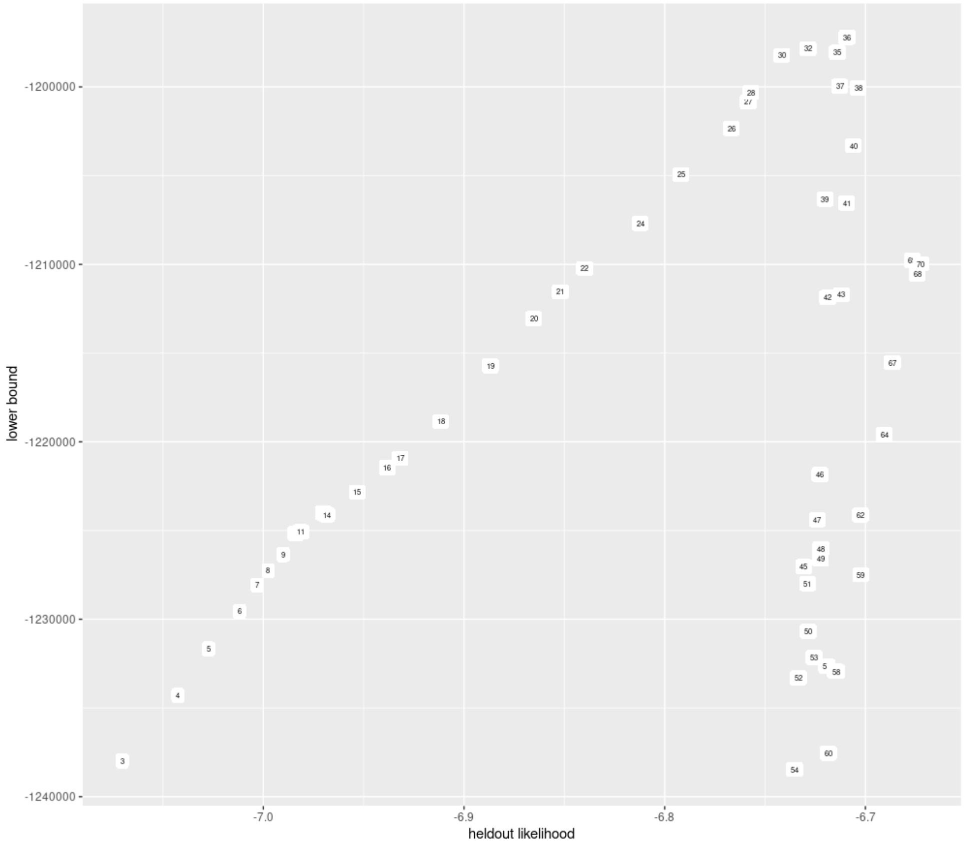

Figure S4: Mean values of *Lower bound* and *Heldout likelihood* (based on 50 replications) of estimated STM different values of K. Labels represent the number of topics K.
At each replication a different random sample containing a 25% of the population of no-deal effects has been heldout. The heldout sample was hence used to compute the *Heldout likelihood* .

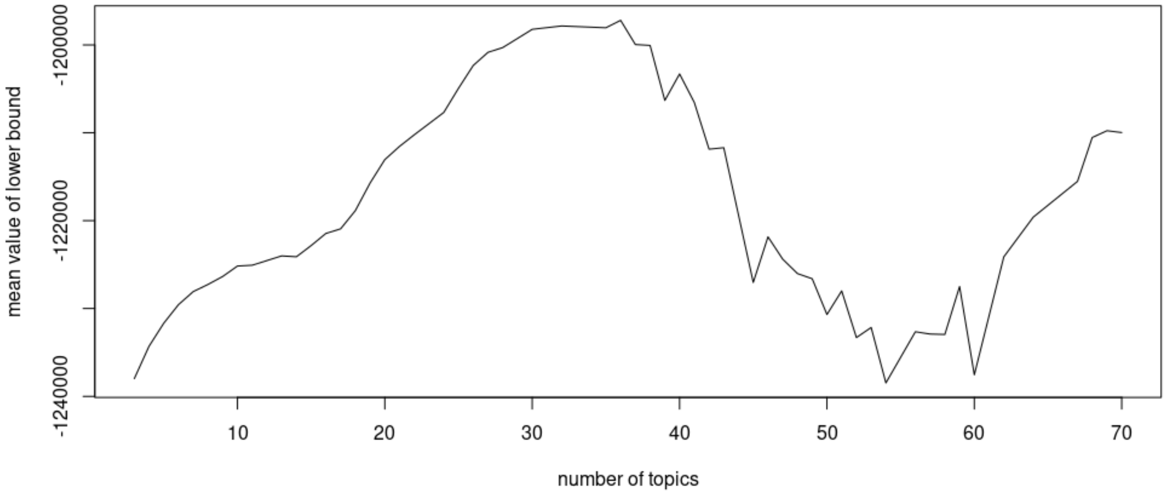

Figure S5: Mean values of the STM *Lower bound* as a function of K. For each replication a different random sample containing a 25% of the population of no-deal effects has been heldout.

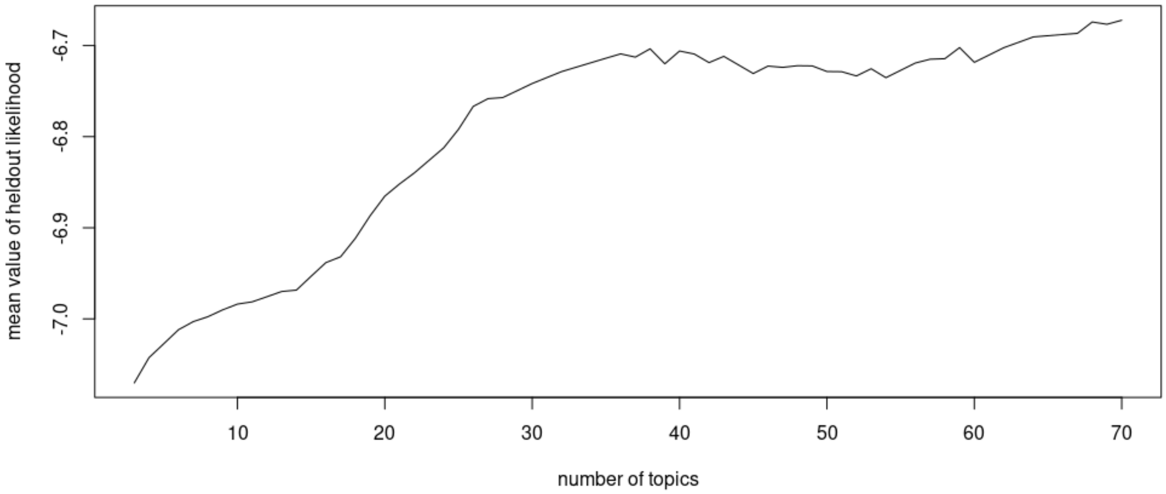

Figure S6: Mean values of the STM. *Heldout likelihood* as a function of K. For each replication a different random sample containing a 25% of the population of no-deal effects has been heldout. The heldout sample was hence used to compute the *Heldout likelihood*.

S3 Methodology

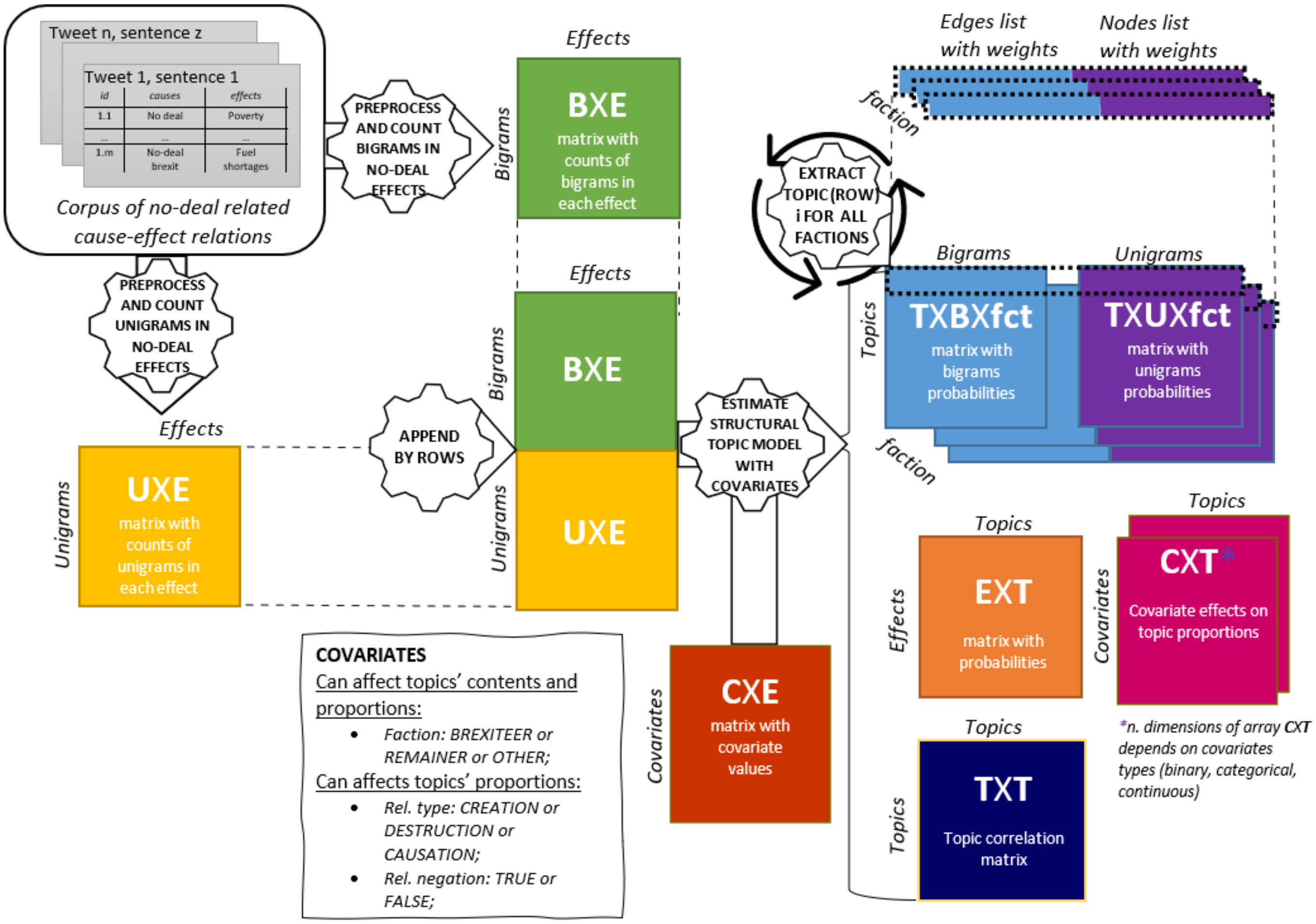

Figure S7: Workflow summary.

**stopwords**
"i", "me", "my", "myself","we","our","ours", "ourselves", "you", "your","yours","yourself", "yourselves","he",<br>"him","his","himself","she","her","hers","herself","it","its","itself","they","them","their", "theirs" ,"themselves",<br>"what","which","who" ,"whom", "this" ,"that" ,"these", "those", "am", "is" ,"are","was","were", "be","been",<br>"being","have" ,"has" ,"had","having","do","does","did","doing","would","should","could","ought","i'm",<br>"you're","he's","she's","it's","we're","they're","i've","you've","we've", "they've","i'd", "you'd", "he'd", "she'd",<br>"we'd", "they'd", "i'll", "you'll", "he'll", "she'll", "we'll", "they'll", "isn't", "aren't", "wasn't", "weren't",<br>"hasn't", "haven't", "hadn't", "doesn't", "don't", "didn't", "won't", "wouldn't" "shan't", "shouldn't" "can't",<br>"cannot", "couldn't" "mustn't", "let's", "that's", "who's", "what's", "here's", "there's", "when's", "where's",<br>"why's", "how's", "a", "an", "the", "and", "but", "because", "as", "until", "while", "of", "at", "by", "for","with",<br>"about", "against", "between", "into", "through", "during", "before", "after", "above", "below", "to", "from",<br>"up", "down", "in", "out", "on", "over", "under", "again", "further", "then", "once", "here", "there", "when",<br>"where", "why", "how", "all", "any", "both", "each", "few", "more", "most", "other", "some", "such", "only",<br>"own", "same", "so", "than", "too", "very", "will", "a", "b", "c", "d", "e", "f", "g", "h", "i", "j", "k", "l",<br>"m", "n", "o", "p", "q", "r", "s", "t", "u", "v", "w", "x", "y", "z", "wa", "us", "ll", "can", "etc", "ve",<br>"imo", "amp", "thatbe", "the", "therebe", "webe", "&", "ie", "via", "also", "think", "simply", "dr",<br>"re", "youwatch" "whomost", "nah", "itbe", "amongst", "lrast", "youto", "theof", "mayhem", "amlo",<br>"oui", "oh", "kap", "ect", "bla", "try", "yet", "thats", "got", "hoc", "lot", "it"

Table S9: List of stopwords (based on Quanteda stopword list) used in STM estimation step.

**RegEx**
([Nn]{1}[Oo]{1}[-_[:space:]]{1}[Dd]{1}[Ee]{1}[Aa]{1}[lL]{1}){1}[\"'?>]{0,1}([[:space:]]+[[A-Za-z_]]+){0,3}[[:space:][:punct:]]{0,}$

Table S10: RegEx used to identify causes with "no-deal" in the last four tokens of the cause-side.

Destruction	*Causation*	*Creation*
destroy/destroys/destroyed/destroying<br>demolish/demolishes/demolished/demolishing<br>annihilate/annihilates/annihilated/annihilating<br>obliterate/obliterates/obliterated/obliterating<br>cancel/cancels/cancelled/canceled/cancelling/canceling<br>eradicate/eradicates/eradicated/eradicating<br>extirpate/extirpates/extirpated/extirpating<br>eliminate/eliminates/eliminated/eliminating<br>wipe out/wipes out/wiped out/wiping out<br>wipe off/wipes off/wiped off/wiping off<br>kill/kills/killed/killing	cause/causes/caused/causing<br>determine/determines/determined/determining<br>engender/engenders/engendered/engendering<br>entail/entails/entailed/entailing<br>provoke/provokes/provoked/provoking<br>trigger/triggers/triggered/triggering<br>leads to/lead to/led to/leading to<br>result in/results in/resulted in/resulting in	create/creates/creating/created<br>produce/produces/produced/producing<br>originate/originates/originated/originating<br>generate/generates/generated/generating<br>make/makes/made/making

Table S11: List of causal verbs employed in RegEx functions by relation verb type (*rel.type*)

Table S12: Relation extraction function and associated regular expressions (R code).

```
\scriptsize
and_regex="([[:punct:][:space:]]){1,}(and also|but also|also|while|
as well as|but([[:punct:][:space:]]){1,}meanwhile|and([[:punct:][:s
pace:]]){1,}meanwhile|meanwhile|but([[:punct:][:space:]]){1,}at the
 same time|and([[:punct:][:space:]]){1,}at the same time|at the sam
e time|and([[:punct:][:space:]]){1,}simultaneously|but([[:punct:][:
space:]]){1,}simultaneously|simultaneously|and([[:punct:][:space:]]
){1,}concurrently|but([[:punct:][:space:]]){1,}concurrently|concurr
ently|and([[:punct:][:space:]]){1,}jointly|but([[:punct:][:space:]]
){1,}jointly|jointly|but|and){1,}([[:punct:][:space:]]){0,}(I|you|h
e|she|it|we|you|they){0,}([[:punct:][:space:]]){0,}$"

all_at_end="create|creates|creating|created|produce|produces|producing|produced
|originate|originates|originating|originated|generate|generates|gen
erated|generating|make|makes|making|made|destroy|destroys|destroyed
|destroying|demolish|demolishes|demolished|demolishing|annihilate|a
nnihilates|annihilated|annihilating|obliterate|obliterates|oblitera
ted|obliterating|cancel|cancels|cancelled|canceled|cancelling|cance
ling|eradicate|eradicates|eradicated|eradicating|extirpate|extirpat
es|extirpated|extirpating|eliminate|eliminates|eliminated|eliminati
ng|wipe out|wipes out|wiped out|wiping out|wipe off|wipes off|wiped
 off|wiping off|kill|kills|killed|killing|cause|causes|causing|caus
ed|determine|determines|determining|determined|engender|engenders|e
ngendered|engendering|entail|entails|entailed|entailing|provoke|pro
vokes|provoked|provoking|trigger|triggers|triggered|triggering"

patterns="(?<![\\pL\\pM_-])(create)(?![\\pL\\pM_-])|(?<![\\pL\\pM_-])(creates)
(?![\\pL\\pM_-])|(?<![\\pL\\pM_-])(creating)(?![\\pL\\pM_-])|
(?<![\\pL\\pM_-])(created)(?![\\pL\\pM_-])|(?<![\\pL\\pM_-])(produce)
(?![\\pL\\pM_-])|(?<![\\pL\\pM_-])(produces)(?![\\pL\\pM_-])|
(?<![\\pL\\pM_-])(producing)(?![\\pL\\pM_-])|(?<![\\pL\\pM_-])(produced)
(?![\\pL\\pM_-])|(?<![\\pL\\pM_-])(originate)(?![\\pL\\pM_
-])|(?<![\\pL\\pM_-])(originates)(?![\\pL\\pM_-])|(?<![\\pL\\pM_-]
)(originating)(?![\\pL\\pM_-])|(?<![\\pL\\pM_-])(originated)(?![\\
pL\\pM_-])|(?<![\\pL\\pM_-])(generate)(?![\\pL\\pM_-])|(?<![\\pL\\p
M_-])(generates)(?![\\pL\\pM_-])|(?<![\\pL\\pM_-])(generated)(?![\
\pL\\pM_-])|(?<![\\pL\\pM_-])(generating)(?![\\pL\\pM_-])|(?<![\\p
L\\pM_-])(make)(?![\\pL\\pM_-])|(?<![\\pL\\pM_-])(makes)(?![\\pL\\
pM_-])|(?<![\\pL\\pM_-])(making)(?![\\pL\\pM_-])|(?<![\\pL\\pM_-])
(made)(?![\\pL\\pM_-])|(?<![\\pL\\pM_-])(destroy)(?![\\pL\\pM_-])|(
?<![\\pL\\pM_-])(destroys)(?![\\pL\\pM_-])|(?<![\\pL\\pM_-])(destro
yed)(?![\\pL\\pM_-])|(?<![\\pL\\pM_-])(destroying)(?![\\pL\\pM_-])|
(?<![\\pL\\pM_-])(demolish)(?![\\pL\\pM_-])|(?<![\\pL\\pM_-])(demol
ishes)(?![\\pL\\pM_-])|(?<![\\pL\\pM_-])(demolished)(?![\\pL\\pM_-]
)|(?<![\\pL\\pM_-])(demolishing)(?![\\pL\\pM_-])|(?<![\\pL\\pM_-])(
annihilate)(?![\\pL\\pM_-])|(?<![\\pL\\pM_-])(annihilates)(?![\\pL\
\pM_-])|(?<![\\pL\\pM_-])(annihilated)(?![\\pL\\pM_-])|(?<![\\pL\\p
M_-])(annihilating)(?![\\pL\\pM_-])|(?<![\\pL\\pM_-])(obliterate)(?
![\\pL\\pM_-])|(?<![\\pL\\pM_-])(obliterates)(?![\\pL\\pM_-])|(?<![
\\pL\\pM_-])(obliterated)(?![\\pL\\pM_-])|(?<![\\pL\\pM_-])(obliter
ating)(?![\\pL\\pM_-])|(?<![\\pL\\pM_-])(cancel)(?![\\pL\\pM_-])|(?
<![\\pL\\pM_-])(cancels)(?![\\pL\\pM_-])|(?<![\\pL\\pM_-])(cancelle
d)(?![\\pL\\pM_-])|(?<![\\pL\\pM_-])(canceled)(?![\\pL\\pM_-])|(?<!
[\\pL\\pM_-])(cancelling)(?![\\pL\\pM_-])|(?<![\\pL\\pM_-])(canceli
ng)(?![\\pL\\pM_-])|(?<![\\pL\\pM_-])(eradicate)(?![\\pL\\pM_-])|(?
<![\\pL\\pM_-])(eradicates)(?![\\pL\\pM_-])|(?<![\\pL\\pM_-])(eradi
cated)(?![\\pL\\pM_-])|(?<![\\pL\\pM_-])(eradicating)(?![\\pL\\pM_-
])|(?<![\\pL\\pM_-])(extirpate)(?![\\pL\\pM_-])|(?<![\\pL\\pM_-])(e
```

```
xtirpates)(?![\\pL\\pM_-])|(?<![\\pL\\pM_-])(extirpated)(?![\\pL\\p
M_-])|(?<![\\pL\\pM_-])(extirpating)(?![\\pL\\pM_-])|(?<![\\pL\\pM_
-])(eliminate)(?![\\pL\\pM_-])|(?<![\\pL\\pM_-])(eliminates)(?![\\p
L\\pM_-])|(?<![\\pL\\pM_-])(eliminated)(?![\\pL\\pM_-])|(?<![\\pL\\
pM_-])(eliminating)(?![\\pL\\pM_-])|(?<![\\pL\\pM_-])(wipe out)(?![
\\pL\\pM_-])|(?<![\\pL\\pM_-])(wipes out)(?![\\pL\\pM_-])|(?<![\\pL
\\pM_-])(wiped out)(?![\\pL\\pM_-])|(?<![\\pL\\pM_-])(wiping out)(?
![\\pL\\pM_-])|(?<![\\pL\\pM_-])(wipe off)(?![\\pL\\pM_-])|(?<![\\p
L\\pM_-])(wipes off)(?![\\pL\\pM_-])|(?<![\\pL\\pM_-])(wiped off)(?
![\\pL\\pM_-])|(?<![\\pL\\pM_-])(wiping off)(?![\\pL\\pM_-])|(?<![\
\pL\\pM_-])(kill)(?![\\pL\\pM_-])|(?<![\\pL\\pM_-])(kills)(?![\\pL\
\pM_-])|(?<![\\pL\\pM_-])(killed)(?![\\pL\\pM_-])|(?<![\\pL\\pM_-])
(killing)(?![\\pL\\pM_-])|(?<![\\pL\\pM_-])(cause)(?![\\pL\\pM_-])|
(?<![\\pL\\pM_-])(causes)(?![\\pL\\pM_-])|(?<![\\pL\\pM_-])(causing
)(?![\\pL\\pM_-])|(?<![\\pL\\pM_-])(caused)(?![\\pL\\pM_-])|(?<![\\
pL\\pM_-])(determine)(?![\\pL\\pM_-])|(?<![\\pL\\pM_-])(determines)
(?![\\pL\\pM_-])|(?<![\\pL\\pM_-])(determining)(?![\\pL\\pM_-])|(?<
![\\pL\\pM_-])(determined)(?![\\pL\\pM_-])|(?<![\\pL\\pM_-])(engend
er)(?![\\pL\\pM_-])|(?<![\\pL\\pM_-])(engenders)(?![\\pL\\pM_-])|(?
<![\\pL\\pM_-])(engendered)(?![\\pL\\pM_-])|(?<![\\pL\\pM_-])(engen
dering)(?![\\pL\\pM_-])|(?<![\\pL\\pM_-])(entail)(?![\\pL\\pM_-])|(
?<![\\pL\\pM_-])(entails)(?![\\pL\\pM_-])|(?<![\\pL\\pM_-])(entaile
d)(?![\\pL\\pM_-])|(?<![\\pL\\pM_-])(entailing)(?![\\pL\\pM_-])|(?<
![\\pL\\pM_-])(provoke)(?![\\pL\\pM_-])|(?<![\\pL\\pM_-])(provokes)
(?![\\pL\\pM_-])|(?<![\\pL\\pM_-])(provoked)(?![\\pL\\pM_-])|(?<![\
\pL\\pM_-])(provoking)(?![\\pL\\pM_-])|(?<![\\pL\\pM_-])(trigger)(?
![\\pL\\pM_-])|(?<![\\pL\\pM_-])(triggers)(?![\\pL\\pM_-])|(?<![\\p
L\\pM_-])(triggered)(?![\\pL\\pM_-])|(?<![\\pL\\pM_-])(triggering)(
?![\\pL\\pM_-])|(?<![\\pL\\pM_-])(leads to)(?![\\pL\\pM_-])|(?<![\\
pL\\pM_-])(lead to)(?![\\pL\\pM_-])|(?<![\\pL\\pM_-])(led to)(?![\\
pL\\pM_-])|(?<![\\pL\\pM_-])(leading to)(?![\\pL\\pM_-])|(?<![\\pL\
\pM_-])(reason of)(?![\\pL\\pM_-])|(?<![\\pL\\pM_-])(result in)(?![
\\pL\\pM_-])|(?<![\\pL\\pM_-])(results in)(?![\\pL\\pM_-])|(?<![\\p
L\\pM_-])(resulted in)(?![\\pL\\pM_-])|(?<![\\pL\\pM_-])(resulting
in)(?![\\pL\\pM_-])"

passive_pattern="(?<![\\pL\\pM_-])(created by
)(?![\\pL\\pM_-])|(?<![\\pL\\pM_-])(produced by)(?![\\pL\\pM_-])|(?
<![\\pL\\pM_-])(originated by)(?![\\pL\\pM_-])|(?<![\\pL\\pM_-])(ge
nerated by)(?![\\pL\\pM_-])|(?<![\\pL\\pM_-])(made by)(?![\\pL\\pM_
-])|(?<![\\pL\\pM_-])(destroyed by)(?![\\pL\\pM_-])|(?<![\\pL\\pM_-
])(demolished by)(?![\\pL\\pM_-])|(?<![\\pL\\pM_-])(annihilated by)
(?![\\pL\\pM_-])|(?<![\\pL\\pM_-])(obliterated by)(?![\\pL\\pM_-])|
(?<![\\pL\\pM_-])(cancelled by)(?![\\pL\\pM_-])|(?<![\\pL\\pM_-])(c
anceled by)(?![\\pL\\pM_-])|(?<![\\pL\\pM_-])(eradicated by)(?![\\p
L\\pM_-])|(?<![\\pL\\pM_-])(extirpated by)(?![\\pL\\pM_-])|(?<![\\p
L\\pM_-])(eliminated by)(?![\\pL\\pM_-])|(?<![\\pL\\pM_-])(wiped ou
t by)(?![\\pL\\pM_-])|(?<![\\pL\\pM_-])(wiped off by)(?![\\pL\\pM_-
])|(?<![\\pL\\pM_-])(killed by)(?![\\pL\\pM_-])|(?<![\\pL\\pM_-])(c
aused by)(?![\\pL\\pM_-])|(?<![\\pL\\pM_-])(determined by)(?![\\pL\
\pM_-])|(?<![\\pL\\pM_-])(engendered by)(?![\\pL\\pM_-])|(?<![\\pL\
\pM_-])(entailed by)(?![\\pL\\pM_-])|(?<![\\pL\\pM_-])(provoked by)
(?![\\pL\\pM_-])|(?<![\\pL\\pM_-])(triggered by)(?![\\pL\\pM_-])|(?
<![\\pL\\pM_-])(cause was)(?![\\pL\\pM_-])|(?<![\\pL\\pM_-])(determ
ine was)(?![\\pL\\pM_-])|(?<![\\pL\\pM_-])(provoke was)(?![\\pL\\pM
_-])|(?<![\\pL\\pM_-])(due to)(?![\\pL\\pM_-])|(?<![\\pL\\pM_-])(le
d by)(?![\\pL\\pM_-])|(?<![\\pL\\pM_-])(result from)(?![\\pL\\pM_-]
)|(?<![\\pL\\pM_-])(results from)(?![\\pL\\pM_-])|(?<![\\pL\\pM_-])
(resulted from)(?![\\pL\\pM_-])|(?<![\\pL\\pM_-])(resulting from)(?
![\\pL\\pM_-])|(?<![\\pL\\pM_-])(stem from)(?![\\pL\\pM_-])|(?<![\\
pL\\pM_-])(stems from)(?![\\pL\\pM_-])|(?<![\\pL\\pM_-])(stemmed fr
om)(?![\\pL\\pM_-])|(?<![\\pL\\pM_-])(stemming from)(?![\\pL\\pM_-]
```

```
)|(?<![\\pL\\pM_-])(derive from)(?![\\pL\\pM_-])|(?<![\\pL\\pM_-])(
derives from)(?![\\pL\\pM_-])|(?<![\\pL\\pM_-])(derived from)(?![\\
pL\\pM_-])|(?<![\\pL\\pM_-])(deriving from)(?![\\pL\\pM_-])|(?<![\\
pL\\pM_-])(originate from)(?![\\pL\\pM_-])|(?<![\\pL\\pM_-])(origin
ates from)(?![\\pL\\pM_-])|(?<![\\pL\\pM_-])(originated from)(?![\\
pL\\pM_-])|(?<![\\pL\\pM_-])(originating from)(?![\\pL\\pM_-])"

pattern_match="[^\\pL\\pM_-]create[^\\pL\\pM_-]|^create[^\\pL\\pM_-]|
[^\\pL\\pM_-]create$|^create$|[^\\pL\\pM_-]creates[^\\pL\\pM_-]|^cr
eates[^\\pL\\pM_-]|[^\\pL\\pM_-]creates$|^creates$|[^\\pL\\pM_-]cre
ating[^\\pL\\pM_-]|^creating[^\\pL\\pM_-]|[^\\pL\\pM_-]creating$|^c
reating$|[^\\pL\\pM_-]created[^\\pL\\pM_-]|^created[^\\pL\\pM_-]|[^
\\pL\\pM_-]created$|^created$|[^\\pL\\pM_-]produce[^\\pL\\pM_-]|^pr
oduce[^\\pL\\pM_-]|[^\\pL\\pM_-]produce$|^produce$|[^\\pL\\pM_-]pro
duces[^\\pL\\pM_-]|^produces[^\\pL\\pM_-]|[^\\pL\\pM_-]produces$|^p
roduces$|[^\\pL\\pM_-]producing[^\\pL\\pM_-]|^producing[^\\pL\\pM_-
]|[^\\pL\\pM_-]producing$|^producing$|[^\\pL\\pM_-]produced[^\\pL\\
pM_-]|^produced[^\\pL\\pM_-]|[^\\pL\\pM_-]produced$|^produced$|[^\\
pL\\pM_-]originate[^\\pL\\pM_-]|^originate[^\\pL\\pM_-]|[^\\pL\\pM_
-]originate$|^originate$|[^\\pL\\pM_-]originates[^\\pL\\pM_-]|^orig
inates[^\\pL\\pM_-]|[^\\pL\\pM_-]originates$|^originates$|[^\\pL\\p
M_-]originating[^\\pL\\pM_-]|^originating[^\\pL\\pM_-]|[^\\pL\\pM_-
]originating$|^originating$|[^\\pL\\pM_-]originated[^\\pL\\pM_-]|^o
riginated[^\\pL\\pM_-]|[^\\pL\\pM_-]originated$|^originated$|[^\\pL
\\pM_-]generate[^\\pL\\pM_-]|^generate[^\\pL\\pM_-]|[^\\pL\\pM_-]ge
nerate$|^generate$|[^\\pL\\pM_-]generates[^\\pL\\pM_-]|^generates[^
\\pL\\pM_-]|[^\\pL\\pM_-]generates$|^generates$|[^\\pL\\pM_-]genera
ted[^\\pL\\pM_-]|^generated[^\\pL\\pM_-]|[^\\pL\\pM_-]generated$|^g
enerated$|[^\\pL\\pM_-]generating[^\\pL\\pM_-]|^generating[^\\pL\\p
M_-]|[^\\pL\\pM_-]generating$|^generating$|[^\\pL\\pM_-]make[^\\pL\
\pM_-]|^make[^\\pL\\pM_-]|[^\\pL\\pM_-]make$|^make$|[^\\pL\\pM_-]ma
kes[^\\pL\\pM_-]|^makes[^\\pL\\pM_-]|[^\\pL\\pM_-]makes$|^makes$|[^
\\pL\\pM_-]making[^\\pL\\pM_-]|^making[^\\pL\\pM_-]|[^\\pL\\pM_-]ma
king$|^making$|[^\\pL\\pM_-]made[^\\pL\\pM_-]|^made[^\\pL\\pM_-]|[^
\\pL\\pM_-]made$|^made$|[^\\pL\\pM_-]destroy[^\\pL\\pM_-]|^destroy[
^\\pL\\pM_-]|[^\\pL\\pM_-]destroy$|^destroy$|[^\\pL\\pM_-]destroys[
^\\pL\\pM_-]|^destroys[^\\pL\\pM_-]|[^\\pL\\pM_-]destroys$|^destroy
s$|[^\\pL\\pM_-]destroyed[^\\pL\\pM_-]|^destroyed[^\\pL\\pM_-]|[^\\
pL\\pM_-]destroyed$|^destroyed$|[^\\pL\\pM_-]destroying[^\\pL\\pM_-
]|^destroying[^\\pL\\pM_-]|[^\\pL\\pM_-]destroying$|^destroying$|[^
\\pL\\pM_-]demolish[^\\pL\\pM_-]|^demolish[^\\pL\\pM_-]|[^\\pL\\pM_
-]demolish$|^demolish$|[^\\pL\\pM_-]demolishes[^\\pL\\pM_-]|^demoli
shes[^\\pL\\pM_-]|[^\\pL\\pM_-]demolishes$|^demolishes$|[^\\pL\\pM_
-]demolished[^\\pL\\pM_-]|^demolished[^\\pL\\pM_-]|[^\\pL\\pM_-]dem
olished$|^demolished$|[^\\pL\
\pM_-]demolishing[^\\pL\\pM_-]|^demolishing[^\\pL\\pM_-]|[^\\pL\\pM
_-]demolishing$|^demolishing$|[^\\pL\\pM_-]annihilate[^\\pL\\pM_-]|
^annihilate[^\\pL\\pM_-]|[^\\pL\\pM_-]annihilate$|^annihilate$|[^\\
pL\\pM_-]annihilates[^\\pL\\pM_-]|^annihilates[^\\pL\\pM_-]|[^\\pL\
\pM_-]annihilates$|^annihilates$|[^\\pL\\pM_-]annihilated[^\\pL\\pM
_-]|^annihilated[^\\pL\\pM_-]|[^\\pL\\pM_-]annihilated$|^annihilate
d$|[^\\pL\\pM_-]annihilating[^\\pL\\pM_-]|^annihilating[^\\pL\\pM_-
]|[^\\pL\\pM_-]annihilating$|^annihilating$|[^\\pL\\pM_-]obliterate
[^\\pL\\pM_-]|^obliterate[^\\pL\\pM_-]|[^\\pL\\pM_-]obliterate$|^ob
literate$|[^\\pL\\pM_-]obliterates[^\\pL\\pM_-]|^obliterates[^\\pL\
\pM_-]|[^\\pL\\pM_-]obliterates$|^obliterates$|[^\\pL\\pM_-]obliter
ated[^\\pL\\pM_-]|^obliterated[^\\pL\\pM_-]|[^\\pL\\pM_-]obliterate
d$|^obliterated$|[^\\pL\\pM_-]obliterating[^\\pL\\pM_-]|^obliterati
ng[^\\pL\\pM_-]|[^\\pL\\pM_-]obliterating$|^obliterating$|[^\\pL\\p
M_-]cancel[^\\pL\\pM_-]|^cancel[^\\pL\\pM_-]|[^\\pL\\pM_-]cancel$|^
cancel$|[^\\pL\\pM_-]cancels[^\\pL\\pM_-]|^cancels[^\\pL\\pM_-]|[^\
\pL\\pM_-]cancels$|^cancels$|[^\\pL\\pM_-]cancelled[^\\pL\\pM_-]|^c
```

```
ancelled[^\\pL\\pM_-]|[^\\pL\\pM_-]cancelled$|^cancelled$|[^\\pL\\p
M_-]canceled[^\\pL\\pM_-]|^canceled[^\\pL\\pM_-]|[^\\pL\\pM_-]cance
led$|^canceled$|[^\\pL\\pM_-]cancelling[^\\pL\\pM_-]|^cancelling[^\
\pL\\pM_-]|[^\\pL\\pM_-]cancelling$|^cancelling$|[^\\pL\\pM_-]cance
ling[^\\pL\\pM_-]|^canceling[^\\pL\\pM_-]|[^\\pL\\pM_-]canceling$|^
canceling$|[^\\pL\\pM_-]eradicate[^\\pL\\pM_-]|^eradicate[^\\pL\\pM
_-]|[^\\pL\\pM_-]eradicate$|^eradicate$|[^\\pL\\pM_-]eradicates[^\\
pL\\pM_-]|^eradicates[^\\pL\\pM_-]|[^\\pL\\pM_-]eradicates$|^eradic
ates$|[^\\pL\\pM_-]eradicated[^\\pL\\pM_-]|^eradicated[^\\pL\\pM_-]
|[^\\pL\\pM_-]eradicated$|^eradicated$|[^\\pL\\pM_-]eradicating[^\\
pL\\pM_-]|^eradicating[^\\pL\\pM_-]|[^\\pL\\pM_-]eradicating$|^erad
icating$|[^\\pL\\pM_-]extirpate[^\\pL\\pM_-]|^extirpate[^\\pL\\pM_-
]|[^\\pL\\pM_-]extirpate$|^extirpate$|[^\\pL\\pM_-]extirpates[^\\pL
\\pM_-]|^extirpates[^\\pL\\pM_-]|[^\\pL\\pM_-]extirpates$|^extirpat
es$|[^\\pL\\pM_-]extirpated[^\\pL\\pM_-]|^extirpated[^\\pL\\pM_-]|[
^\\pL\\pM_-]extirpated$|^extirpated$|[^\\pL\\pM_-]extirpating[^\\pL
\\pM_-]|^extirpating[^\\pL\\pM_-]|[^\\pL\\pM_-]extirpating$|^extirp
ating$|[^\\pL\\pM_-]eliminate[^\\pL\\pM_-]|^eliminate[^\\pL\\pM_-]|
[^\\pL\\pM_-]eliminate$|^eliminate$|[^\\pL\\pM_-]eliminates[^\\pL\\
pM_-]|^eliminates[^\\pL\\pM_-]|[^\\pL\\pM_-]eliminates$|^eliminates
$|[^\\pL\\pM_-]eliminated[^\\pL\\pM_-]|^eliminated[^\\pL\\pM_-]|[^\
\pL\\pM_-]eliminated$|^eliminated$|[^\\pL\\pM_-]eliminating[^\\pL\\
pM_-]|^eliminating[^\\pL\\pM_-]|[^\\pL\\pM_-]eliminating$|^eliminat
ing$|[^\\pL\\pM_-]wipe out[^\\pL\\pM_-]|^wipe out[^\\pL\\pM_-]|[^\\
pL\\pM_-]wipe out$|^wipe out$|[^\\pL\\pM_-]wipes out[^\\pL\\pM_-]|^
wipes out[^\\pL\\pM_-]|[^\\pL\\pM_-]wipes out$|^wipes out$|[^\\pL\\
pM_-]wiped out[^\\pL\\pM_-]|^wiped out[^\\pL\\pM_-]|[^\\pL\\pM_-]wi
ped out$|^wiped out$|[^\\pL\\pM_-]wiping out[^\\pL\\pM_-]|^wiping o
ut[^\\pL\\pM_-]|[^\\pL\\pM_-]wiping out$|^wiping out$|[^\\pL\\pM_-]
wipe off[^\\pL\\pM_-]|^wipe off[^\\pL\\pM_-]|[^\\pL\\pM_-]wipe off$
|^wipe off$|[^\\pL\\pM_-]wipes off[^\\pL\\pM_-]|^wipes off[^\\pL\\p
M_-]|[^\\pL\\pM_-]wipes off$|^wipes off$|[^\\pL\\pM_-]wiped off[^\\
pL\\pM_-]|^wiped off[^\\pL\\pM_-]|[^\\pL\\pM_-]wiped off$|^wiped of
f$|[^\\pL\\pM_-]wiping off[^\\pL\\pM_-]|^wiping off[^\\pL\\pM_-]|[^
\\pL\\pM_-]wiping off$|^wiping off$|[^\\pL\\pM_-]kill[^\\pL\\pM_-]|
^kill[^\\pL\\pM_-]|[^\\pL\\pM_-]kill$|^kill$|[^\\pL\\pM_-]kills[^\\
pL\\pM_-]|^kills[^\\pL\\pM_-]|[^\\pL\\pM_-]kills$|^kills$|[^\\pL\\p
M_-]killed[^\\pL\\pM_-]|^killed[^\\pL\\pM_-]|[^\\pL\\pM_-]killed$|^
killed$|[^\\pL\\pM_-]killing[^\\pL\\pM_-]|^killing[^\\pL\\pM_-]|[^\
\pL\\pM_-]killing$|^killing$|[^\\pL\\pM_-]cause[^\\pL\\pM_-]|^cause
[^\\pL\\pM_-]|[^\\pL\\pM_-]cause$|^cause$|[^\\pL\\pM_-]causes[^\\pL
\\pM_-]|^causes[^\\pL\\pM_-]|[^\\pL\\pM_-]causes$|^causes$|[^\\pL\\
pM_-]causing[^\\pL\\pM_-]|^causing[^\\pL\\pM_-]|[^\\pL\\pM_-]causin
g$|^causing$|[^\\pL\\pM_-]caused[^\\pL\\pM_-]|^caused[^\\pL\\pM_-]|
[^\\pL\\pM_-]caused$|^caused$|[^\\pL\\pM_-]determine[^\\pL\\pM_-]|^
determine[^\\pL\\pM_-]|[^\\pL\\pM_-]determine$|^determine$|[^\\pL\\
pM_-]determines[^\\pL\\pM_-]|^determines[^\\pL\\pM_-]|[^\\pL\\pM_-]
determines$|^determines$|[^\\pL\\pM_-]determining[^\\pL\\pM_-]|^det
ermining[^\\pL\\pM_-]|[^\\pL\\pM_-]determining$|^determining$|[^\\p
L\\pM_-]determined[^\\pL\\pM_-]|^determined[^\\pL\\pM_-]|[^\\pL\\pM
_-]determined$|^determined$|[^\\pL\\pM_-]engender[^\\pL\\pM_-]|^eng
ender[^\\pL\\pM_-]|[^\\pL\\pM_-]engender$|^engender$|[^\\pL\\pM_-]e
ngenders[^\\pL\\pM_-]|^engenders[^\\pL\\pM_-]|[^\\pL\\pM_-]engender
s$|^engenders$|[^\\pL\\pM_-]engendered[^\\pL\\pM_-]|^engendered[^\\
pL\\pM_-]|[^\\pL\\pM_-]engendered$|^engendered$|[^\\pL\\pM_-]engend
ering[^\\pL\\pM_-]|^engendering[^\\pL\\pM_-]|[^\\pL\\pM_-]engenderi
ng$|^engendering$|[^\\pL\\pM_-]entail[^\\pL\\pM_-]|^entail[^\\pL\\p
M_-]|[^\\pL\\pM_-]entail$|^entail$|[^\\pL\\pM_-]entails[^\\pL\\pM_-
]|^entails[^\\pL\\pM_-]|[^\\pL\\pM_-]entails$|^entails$|[^\\pL\\pM_
-]entailed[^\\pL\\pM_-]|^entailed[^\\pL\\pM_-]|[^\\pL\\pM_-]entaile
d$|^entailed$|[^\\pL\\pM_-]ent
ailing[^\\pL\\pM_-]|^entailing[^\\pL\\pM_-]|[^\\pL\\pM_-]entailing$
```

```
|^entailing$|[^\\pL\\pM_-]provoke[^\\pL\\pM_-]|^provoke[^\\pL\\pM_-
]|[^\\pL\\pM_-]provoke$|^provoke$|[^\\pL\\pM_-]provokes[^\\pL\\pM_-
]|^provokes[^\\pL\\pM_-]|[^\\pL\\pM_-]provokes$|^provokes$|[^\\pL\\
pM_-]provoked[^\\pL\\pM_-]|^provoked[^\\pL\\pM_-]|[^\\pL\\pM_-]prov
oked$|^provoked$|[^\\pL\\pM_-]provoking[^\\pL\\pM_-]|^provoking[^\\
pL\\pM_-]|[^\\pL\\pM_-]provoking$|^provoking$|[^\\pL\\pM_-]trigger[
^\\pL\\pM_-]|^trigger[^\\pL\\pM_-]|[^\\pL\\pM_-]trigger$|^trigger$|
[^\\pL\\pM_-]triggers[^\\pL\\pM_-]|^triggers[^\\pL\\pM_-]|[^\\pL\\p
M_-]triggers$|^triggers$|[^\\pL\\pM_-]triggered[^\\pL\\pM_-]|^trigg
ered[^\\pL\\pM_-]|[^\\pL\\pM_-]triggered$|^triggered$|[^\\pL\\pM_-]
triggering[^\\pL\\pM_-]|^triggering[^\\pL\\pM_-]|[^\\pL\\pM_-]trigg
ering$|^triggering$|[^\\pL\\pM_-]leads to[^\\pL\\pM_-]|^leads to[^\
\pL\\pM_-]|[^\\pL\\pM_-]leads to$|^leads to$|[^\\pL\\pM_-]lead to[^
\\pL\\pM_-]|^lead to[^\\pL\\pM_-]|[^\\pL\\pM_-]lead to$|^lead to$|[
^\\pL\\pM_-]led to[^\\pL\\pM_-]|^led to[^\\pL\\pM_-]|[^\\pL\\pM_-]l
ed to$|^led to$|[^\\pL\\pM_-]leading to[^\\pL\\pM_-]|^leading to[^\
\pL\\pM_-]|[^\\pL\\pM_-]leading to$|^leading to$|[^\\pL\\pM_-]reaso
n of[^\\pL\\pM_-]|^reason of[^\\pL\\pM_-]|[^\\pL\\pM_-]reason of$|^
reason of$|[^\\pL\\pM_-]result in[^\\pL\\pM_-]|^result in[^\\pL\\pM
_-]|[^\\pL\\pM_-]result in$|^result in$|[^\\pL\\pM_-]results in[^\\
pL\\pM_-]|^results in[^\\pL\\pM_-]|[^\\pL\\pM_-]results in$|^result
s in$|[^\\pL\\pM_-]resulted in[^\\pL\\pM_-]|^resulted in[^\\pL\\pM_
-]|[^\\pL\\pM_-]resulted in$|^resulted in$|[^\\pL\\pM_-]resulting i
n[^\\pL\\pM_-]|^resulting in[^\\pL\\pM_-]|[^\\pL\\pM_-]resulting in
$|^resulting in$|[^\\pL\\pM_-]due to[^\\pL\\pM_-]|^due to[^\\pL\\pM
_-]|[^\\pL\\pM_-]due to$|^due to$|[^\\pL\\pM_-]led by[^\\pL\\pM_-]|
^led by[^\\pL\\pM_-]|[^\\pL\\pM_-]led by$|^led by$|[^\\pL\\pM_-]res
ult from[^\\pL\\pM_-]|^result from[^\\pL\\pM_-]|[^\\pL\\pM_-]result
 from$|^result from$|[^\\pL\\pM_-]results from[^\\pL\\pM_-]|^result
s from[^\\pL\\pM_-]|[^\\pL\\pM_-]results from$|^results from$|[^\\p
L\\pM_-]resulted from[^\\pL\\pM_-]|^resulted from[^\\pL\\pM_-]|[^\\
pL\\pM_-]resulted from$|^resulted from$|[^\\pL\\pM_-]resulting from
[^\\pL\\pM_-]|^resulting from[^\\pL\\pM_-]|[^\\pL\\pM_-]resulting f
rom$|^resulting from$|[^\\pL\\pM_-]stem from[^\\pL\\pM_-]|^stem fro
m[^\\pL\\pM_-]|[^\\pL\\pM_-]stem from$|^stem from$|[^\\pL\\pM_-]ste
ms from[^\\pL\\pM_-]|^stems from[^\\pL\\pM_-]|[^\\pL\\pM_-]stems fr
om$|^stems from$|[^\\pL\\pM_-]stemmed from[^\\pL\\pM_-]|^stemmed fr
om[^\\pL\\pM_-]|[^\\pL\\pM_-]stemmed from$|^stemmed from$|[^\\pL\\p
M_-]stemming from[^\\pL\\pM_-]|^stemming from[^\\pL\\pM_-]|[^\\pL\\
pM_-]stemming from$|^stemming from$|[^\\pL\\pM_-]derive from[^\\pL\
\pM_-]|^derive from[^\\pL\\pM_-]|[^\\pL\\pM_-]derive from$|^derive
from$|[^\\pL\\pM_-]derives from[^\\pL\\pM_-]|^derives from[^\\pL\\p
M_-]|[^\\pL\\pM_-]derives from$|^derives from$|[^\\pL\\pM_-]derived
 from[^\\pL\\pM_-]|^derived from[^\\pL\\pM_-]|[^\\pL\\pM_-]derived
from$|^derived from$|[^\\pL\\pM_-]deriving from[^\\pL\\pM_-]|^deriv
ing from[^\\pL\\pM_-]|[^\\pL\\pM_-]deriving from$|^deriving from$|[
^\\pL\\pM_-]originate from[^\\pL\\pM_-]|^originate from[^\\pL\\pM_-
]|[^\\pL\\pM_-]originate from$|^originate from$|[^\\pL\\pM_-]origin
ates from[^\\pL\\pM_-]|^originates from[^\\pL\\pM_-]|[^\\pL\\pM_-]o
riginates from$|^originates from$|[^\\pL\\pM_-]originated from[^\\p
L\\pM_-]|^originated from[^\\pL\\pM_-]|[^\\pL\\pM_-]originated from
$|^originated from$|[^\\pL\\pM_-]originating from[^\\pL\\pM_-]|^ori
ginating from[^\\pL\\pM_-]|[^\\pL\\pM_-]originating from$|^originat
ing from$"

pattern_match_all="[^\\pL\\pM_-]create[^\\pL\\pM_-]|^cre
ate[^\\pL\\pM_-]|[^\\pL\\pM_-]create$|^create$|[^\\pL\\pM_-]creates
[^\\pL\\pM_-]|^creates[^\\pL\\pM_-]|[^\\pL\\pM_-]creates$|^creates
$|[^\\pL\\pM_-]creating[^\\pL\\pM_-]|^creating[^\\pL\\pM_-]|[^\\pL\
\pM_-]creating$|^creating$|[^\\pL\\pM_-]created[^\\pL\\pM_-]|^creat
ed[^\\pL\\pM_-]|[^\\pL\\pM_-]created$|^created$|[^\\pL\\pM_-]produc
e[^\\pL\\pM_-]|^produce[^\\pL\\pM_-]|[^\\pL\\pM_-]produce$|^produce
```

```
$|[^\\pL\\pM_-]produces[^\\pL\\pM_-]|^produces[^\\pL\\pM_-]|[^\\pL\
\pM_-]produces$|^produces$|[^\\pL\\pM_-]producing[^\\pL\\pM_-]|^pro
ducing[^\\pL\\pM_-]|[^\\pL\\pM_-]producing$|^producing$|[^\\pL\\pM_
-]produced[^\\pL\\pM_-]|^produced[^\\pL\\pM_-]|[^\\pL\\pM_-]produce
d$|^produced$|[^\\pL\\pM_-]originate[^\\pL\\pM_-]|^originate[^\\pL\
\pM_-]|[^\\pL\\pM_-]originate$|^originate$|[^\\pL\\pM_-]originates[
^\\pL\\pM_-]|^originates[^\\pL\\pM_-]|[^\\pL\\pM_-]originates$|^ori
ginates$|[^\\pL\\pM_-]originating[^\\pL\\pM_-]|^originating[^\\pL\\
pM_-]|[^\\pL\\pM_-]originating$|^originating$|[^\\pL\\pM_-]originat
ed[^\\pL\\pM_-]|^originated[^\\pL\\pM_-]|[^\\pL\\pM_-]originated$|^
originated$|[^\\pL\\pM_-]generate[^\\pL\\pM_-]|^generate[^\\pL\\pM_
-]|[^\\pL\\pM_-]generate$|^generate$|[^\\pL\\pM_-]generates[^\\pL\\
pM_-]|^generates[^\\pL\\pM_-]|[^\\pL\\pM_-]generates$|^generates$|[
^\\pL\\pM_-]generated[^\\pL\\pM_-]|^generated[^\\pL\\pM_-]|[^\\pL\\
pM_-]generated$|^generated$|[^\\pL\\pM_-]generating[^\\pL\\pM_-]|^g
enerating[^\\pL\\pM_-]|[^\\pL\\pM_-]generating$|^generating$|[^\\pL
\\pM_-]make[^\\pL\\pM_-]|^make[^\\pL\\pM_-]|[^\\pL\\pM_-]make$|^mak
e$|[^\\pL\\pM_-]makes[^\\pL\\pM_-]|^makes[^\\pL\\pM_-]|[^\\pL\\pM_-
]makes$|^makes$|[^\\pL\\pM_-]making[^\\pL\\pM_-]|^making[^\\pL\\pM_
-]|[^\\pL\\pM_-]making$|^making$|[^\\pL\\pM_-]made[^\\pL\\pM_-]|^ma
de[^\\pL\\pM_-]|[^\\pL\\pM_-]made$|^made$|[^\\pL\\pM_-]destroy[^\\p
L\\pM_-]|^destroy[^\\pL\\pM_-]|[^\\pL\\pM_-]destroy$|^destroy$|[^\\
pL\\pM_-]destroys[^\\pL\\pM_-]|^destroys[^\\pL\\pM_-]|[^\\pL\\pM_-]
destroys$|^destroys$|[^\\pL\\pM_-]destroyed[^\\pL\\pM_-]|^destroyed
[^\\pL\\pM_-]|[^\\pL\\pM_-]destroyed$|^destroyed$|[^\\pL\\pM_-]dest
roying[^\\pL\\pM_-]|^destroying[^\\pL\\pM_-]|[^\\pL\\pM_-]destroyin
g$|^destroying$|[^\\pL\\pM_-]demolish[^\\pL\\pM_-]|^demolish[^\\pL\
\pM_-]|[^\\pL\\pM_-]demolish$|^demolish$|[^\\pL\\pM_-]demolishes[^\
\pL\\pM_-]|^demolishes[^\\pL\\pM_-]|[^\\pL\\pM_-]demolishes$|^demol
ishes$|[^\\pL\\pM_-]demolished[^\\pL\\pM_-]|^demolished[^\\pL\\pM_-
]|[^\\pL\\pM_-]demolished$|^demolished$|[^\\pL\\pM_-]demolishing[^\
\pL\\pM_-]|^demolishing[^\\pL\\pM_-]|[^\\pL\\pM_-]demolishing$|^dem
olishing$|[^\\pL\\pM_-]annihilate[^\\pL\\pM_-]|^annihilate[^\\pL\\p
M_-]|[^\\pL\\pM_-]annihilate$|^annihilate$|[^\\pL\\pM_-]annihilates
[^\\pL\\pM_-]|^annihilates[^\\pL\\pM_-]|[^\\pL\\pM_-]annihilates$|^
annihilates$|[^\\pL\\pM_-]annihilated[^\\pL\\pM_-]|^annihilated[^\\
pL\\pM_-]|[^\\pL\\pM_-]annihilated$|^annihilated$|[^\\pL\\pM_-]anni
hilating[^\\pL\\pM_-]|^annihilating[^\\pL\\pM_-]|[^\\pL\\pM_-]annih
ilating$|^annihilating$|[^\\pL\\pM_-]obliterate[^\\pL\\pM_-]|^oblit
erate[^\\pL\\pM_-]|[^\\pL\\pM_-]obliterate$|^obliterate$|[^\\pL\\pM
_-]obliterates[^\\pL\\pM_-]|^obliterates[^\\pL\\pM_-]|[^\\pL\\pM_-]
obliterates$|^obliterates$|[^\\pL\\pM_-]obliterated[^\\pL\\pM_-]|^o
bliterated[^\\pL\\pM_-]|[^\\pL\\pM_-]obliterated$|^obliterated$|[^\
\pL\\pM_-]obliterating[^\\pL\\pM_-]|^obliterating[^\\pL\\pM_-]|[^\\
pL\\pM_-]obliterating$|^obliterating$|[^\\pL\\pM_-]cancel[^\\pL\\pM
_-]|^cancel[^\\pL\\pM_-]|[^\\pL\\pM_-]cancel$|^cancel$|[^\\pL\\pM_-
]cancels[^\\pL\\pM_-]|^cancels[^\\pL\\pM_-]|[^\\pL\\pM_-]cancels$|^
cancels$|[^\\pL\\pM_-]cancelled[^\\pL\\pM_-]|^cancelled[^\\pL\\pM_-
]|[^\\pL\\pM_-]cancelled$|^cancelled$|[^\\pL\\pM_-]canceled[^\\pL\\
pM_-]|^canceled[^\\pL\\pM_-]|[^\\pL\\pM_-]canceled$|^canceled$|[^\\
pL\\pM_-]cancelling[^\\pL\\pM_-]|^cancelling[^\\pL\\pM_-]|[^\\pL\\p
M_-]cancelling$|^cancelling$|[^\\pL\\pM_-]canceling[^\\pL\\pM_-]|^c
anceling[^\\pL\\pM_-]|[^\\pL\\pM_-]canceling$|^canceling$|[^\\pL\\p
M_-]eradicate[^\\pL\\pM_-]|^eradicate[^\\pL\\pM_-]|[^\\pL\\pM_-]era
dicate$|^eradicate$|[^\\pL\\pM_-]eradicates[^\\pL\\pM_-]|^eradicate
s[^\\pL\\pM_-]|[^\\pL\\pM_-]eradicates$|^eradicates$|[^\\pL\\pM_-]e
radicated[^\\pL\\pM_-]|^eradicated[^\\pL\\pM_-]|[^\\pL\\pM_-]eradic
ated$|^eradicated$|[^\\pL\\pM_-]eradicating[^\\pL\\pM_-]|^eradicati
ng[^\\pL\\pM_-]|[^\\pL\\pM_-]eradicating$|^eradicating$|[^\\pL\\pM_
-]extirpate[^\\pL\\pM_-]|^extirpate[^\\pL\\pM_-]|[^\\pL\\pM_-]extir
pate$|^extirpate$|[^\\pL\\pM_-]extirpates[^\\pL\\pM_-]|^extirpates[
^\\pL\\pM_-]|[^\\pL\\pM_-]extirpates$|^extirpates$|[^\\pL\\pM_-]ext
```

```
irpated[^\\pL\\pM_-]|^extirpated[^\\pL\\pM_-]|[^\\pL\\pM_-]extirpat
ed$|^extirpated$|[^\\pL\\pM_-]extirpating[^\\pL\\pM_-]|^extirpating
[^\\pL\\pM_-]|[^\\pL\\pM_-]extirpating$|^extirpating$|[^\\pL\\pM_-]
eliminate[^\\pL\\pM_-]|^eliminate[^\\pL\\pM_-]|[^\\pL\\pM_-]elimina
te$|^eliminate$|[^\\pL\\pM_-]eliminates[^\\pL\\pM_-]|^eliminates[^\
\pL\\pM_-]|[^\\pL\\pM_-]eliminates$|^eliminates$|[^\\pL\\pM_-]elimi
nated[^\\pL\\pM_-]|^eliminated[^\\pL\\pM_-]|[^\\pL\\pM_-]eliminated
$|^eliminated$|[^\\pL\\pM_-]eliminating[^\\pL\\pM_-]|^eliminating[^
\\pL\\pM_-]|[^\\pL\\pM_-]eliminating$|^eliminating$|[^\\pL\\pM_-]wipe out[^\\pL\\pM_-]|^wipe out[
^\\pL\\pM_-]|[^\\pL\\pM_-]wipe out$|^wipe out$|[^\\pL\\pM_-]wipes o
ut[^\\pL\\pM_-]|^wipes out[^\\pL\\pM_-]|[^\\pL\\pM_-]wipes out$|^wi
pes out$|[^\\pL\\pM_-]wiped out[^\\pL\\pM_-]|^wiped out[^\\pL\\pM_-
]|[^\\pL\\pM_-]wiped out$|^wiped out$|[^\\pL\\pM_-]wiping out[^\\pL
\\pM_-]|^wiping out[^\\pL\\pM_-]|[^\\pL\\pM_-]wiping out$|^wiping o
ut$|[^\\pL\\pM_-]wipe off[^\\pL\\pM_-]|^wipe off[^\\pL\\pM_-]|[^\\p
L\\pM_-]wipe off$|^wipe off$|[^\\pL\\pM_-]wipes off[^\\pL\\pM_-]|^w
ipes off[^\\pL\\pM_-]|[^\\pL\\pM_-]wipes off$|^wipes off$|[^\\pL\\p
M_-]wiped off[^\\pL\\pM_-]|^wiped off[^\\pL\\pM_-]|[^\\pL\\pM_-]wip
ed off$|^wiped off$|[^\\pL\\pM_-]wiping off[^\\pL\\pM_-]|^wiping of
f[^\\pL\\pM_-]|[^\\pL\\pM_-]wiping off$|^wiping off$|[^\\pL\\pM_-]k
ill[^\\pL\\pM_-]|^kill[^\\pL\\pM_-]|[^\\pL\\pM_-]kill$|^kill$|[^\\p
L\\pM_-]kills[^\\pL\\pM_-]|^kills[^\\pL\\pM_-]|[^\\pL\\pM_-]kills$|
^kills$|[^\\pL\\pM_-]killed[^\\pL\\pM_-]|^killed[^\\pL\\pM_-]|[^\\p
L\\pM_-]killed$|^killed$|[^\\pL\\pM_-]killing[^\\pL\\pM_-]|^killing
[^\\pL\\pM_-]|[^\\pL\\pM_-]killing$|^killing$|[^\\pL\\pM_-]cause[^\
\pL\\pM_-]|^cause[^\\pL\\pM_-]|[^\\pL\\pM_-]cause$|^cause$|[^\\pL\\
pM_-]causes[^\\pL\\pM_-]|^causes[^\\pL\\pM_-]|[^\\pL\\pM_-]causes$|
^causes$|[^\\pL\\pM_-]causing[^\\pL\\pM_-]|^causing[^\\pL\\pM_-]|[^
\\pL\\pM_-]causing$|^causing$|[^\\pL\\pM_-]caused[^\\pL\\pM_-]|^cau
sed[^\\pL\\pM_-]|[^\\pL\\pM_-]caused$|^caused$|[^\\pL\\pM_-]determi
ne[^\\pL\\pM_-]|^determine[^\\pL\\pM_-]|[^\\pL\\pM_-]determine$|^de
termine$|[^\\pL\\pM_-]determines[^\\pL\\pM_-]|^determines[^\\pL\\pM
_-]|[^\\pL\\pM_-]determines$|^determines$|[^\\pL\\pM_-]determining[
^\\pL\\pM_-]|^determining[^\\pL\\pM_-]|[^\\pL\\pM_-]determining$|^d
etermining$|[^\\pL\\pM_-]determined[^\\pL\\pM_-]|^determined[^\\pL\
\pM_-]|[^\\pL\\pM_-]determined$|^determined$|[^\\pL\\pM_-]engender[
^\\pL\\pM_-]|^engender[^\\pL\\pM_-]|[^\\pL\\pM_-]engender$|^engende
r$|[^\\pL\\pM_-]engenders[^\\pL\\pM_-]|^engenders[^\\pL\\pM_-]|[^\\
pL\\pM_-]engenders$|^engenders$|[^\\pL\\pM_-]engendered[^\\pL\\pM_-
]|^engendered[^\\pL\\pM_-]|[^\\pL\\pM_-]engendered$|^engendered$|[^
\\pL\\pM_-]engendering[^\\pL\\pM_-]|^engendering[^\\pL\\pM_-]|[^\\p
L\\pM_-]engendering$|^engendering$|[^\\pL\\pM_-]entail[^\\pL\\pM_-]
|^entail[^\\pL\\pM_-]|[^\\pL\\pM_-]entail$|^entail$|[^\\pL\\pM_-]en
tails[^\\pL\\pM_-]|^entails[^\\pL\\pM_-]|[^\\pL\\pM_-]entails$|^ent
ails$|[^\\pL\\pM_-]entailed[^\\pL\\pM_-]|^entailed[^\\pL\\pM_-]|[^\
\pL\\pM_-]entailed$|^entailed$|[^\\pL\\pM_-]entailing[^\\pL\\pM_-]|
^entailing[^\\pL\\pM_-]|[^\\pL\\pM_-]entailing$|^entailing$|[^\\pL\
\pM_-]provoke[^\\pL\\pM_-]|^provoke[^\\pL\\pM_-]|[^\\pL\\pM_-]provo
ke$|^provoke$|[^\\pL\\pM_-]provokes[^\\pL\\pM_-]|^provokes[^\\pL\\p
M_-]|[^\\pL\\pM_-]provokes$|^provokes$|[^\\pL\\pM_-]provoked[^\\pL\
\pM_-]|^provoked[^\\pL\\pM_-]|[^\\pL\\pM_-]provoked$|^provoked$|[^\
\pL\\pM_-]provoking[^\\pL\\pM_-]|^provoking[^\\pL\\pM_-]|[^\\pL\\pM
_-]provoking$|^provoking$|[^\\pL\\pM_-]trigger[^\\pL\\pM_-]|^trigge
r[^\\pL\\pM_-]|[^\\pL\\pM_-]trigger$|^trigger$|[^\\pL\\pM_-]trigger
s[^\\pL\\pM_-]|^triggers[^\\pL\\pM_-]|[^\\pL\\pM_-]triggers$|^trigg
ers$|[^\\pL\\pM_-]triggered[^\\pL\\pM_-]|^triggered[^\\pL\\pM_-]|[^
\\pL\\pM_-]triggered$|^triggered$|[^\\pL\\pM_-]triggering[^\\pL\\pM
_-]|^triggering[^\\pL\\pM_-]|[^\\pL\\pM_-]triggering$|^triggering$|
[^\\pL\\pM_-]leads to[^\\pL\\pM_-]|^leads to[^\\pL\\pM_-]|[^\\pL\\p
M_-]leads to$|^leads to$|[^\\pL\\pM_-]lead to[^\\pL\\pM_-]|^lead to
[^\\pL\\pM_-]|[^\\pL\\pM_-]lead to$|^lead to$|[^\\pL\\pM_-]led to[^
\\pL\\pM_-]|^led to[^\\pL\\pM_-]|[^\\pL\\pM_-]led to$|^led to$|[^\\
```

```
pL\\pM_-]leading to[^\\pL\\pM_-]|^leading to[^\\pL\\pM_-]|[^\\pL\\p
M_-]leading to$|^leading to$|[^\\pL\\pM_-]reason of[^\\pL\\pM_-]|^r
eason of[^\\pL\\pM_-]|[^\\pL\\pM_-]reason of$|^reason of$|[^\\pL\\p
M_-]result in[^\\pL\\pM_-]|^result in[^\\pL\\pM_-]|[^\\pL\\pM_-]res
ult in$|^result in$|[^\\pL\\pM_-]results in[^\\pL\\pM_-]|^results i
n[^\\pL\\pM_-]|[^\\pL\\pM_-]results in$|^results in$|[^\\pL\\pM_-]r
esulted in[^\\pL\\pM_-]|^resulted in[^\\pL\\pM_-]|[^\\pL\\pM_-]resu
lted in$|^resulted in$|[^\\pL\\pM_-]resulting in[^\\pL\\pM_-]|^resu
lting in[^\\pL\\pM_-]|[^\\pL\\pM_-]resulting in$|^resulting in$|[^\
\pL\\pM_-]due to[^\\pL\\pM_-]|^due to[^\\pL\\pM_-]|[^\\pL\\pM_-]due
 to$|^due to$|[^\\pL\\pM_-]led by[^\\pL\\pM_-]|^led by[^\\pL\\pM_-]
|[^\\pL\\pM_-]led by$|^led by$|[^\\pL\\pM_-]result from[^\\pL\\pM_-
]|^result from[^\\pL\\pM_-]|[^\\pL\\pM_-]result from$|^result from$
|[^\\pL\\pM_-]results from[^\\pL\\pM_-]|^results from[^\\pL\\pM_-]|
[^\\pL\\pM_-]results from$|^results from$|[^\\pL\\pM_-]resulted fro
m[^\\pL\\pM_-]|^resulted from[^\\pL\\pM_-]|[^\\pL\\pM_-]resulted fr
om$|^resulted from$|[^\\pL\\pM_-]resulting from[^\\pL\\pM_-]|^resul
ting from[^\\pL\\pM_-]|[^\\pL\\pM_-]resulting from$|^resulting from
$|[^\\pL\\pM_-]stem from[^\\pL\\pM_-]|^stem from[^\\pL\\pM_-]|[^\\p
L\\pM_-]stem from$|^stem from$|[^\\pL\\pM_-]stems from[^\\pL\\pM_-]
|^stems from[^\\pL\\pM_-]|[^\\pL\\pM_-]stems from$|^stems from$|[^\
\pL\\pM_-]stemmed from[^\\pL\\pM_-]|^stemmed from[^\\pL\\pM_-]|[^\\
pL\\pM_-]stemmed from$|^stemmed from$|[^\\pL\\pM_-]stemming from[^\
\pL\\pM_-]|^stemming from[^\\pL\\pM_-]|[^\\pL\\pM_-]stemming from$|
^stemming from$|[^\\pL\\pM_-]derive from[^\\pL\\pM_-]|^derive from[^\\pL\\pM_-]|[^\\pL\\pM_-]der
ive from$|^derive from$|[^\\pL\\pM_-]derives from[^\\pL\\pM_-]|^der
ives from[^\\pL\\pM_-]|[^\\pL\\pM_-]derives from$|^derives from$|[^
\\pL\\pM_-]derived from[^\\pL\\pM_-]|^derived from[^\\pL\\pM_-]|[^\
\pL\\pM_-]derived from$|^derived from$|[^\\pL\\pM_-]deriving from[^
\\pL\\pM_-]|^deriving from[^\\pL\\pM_-]|[^\\pL\\pM_-]deriving from$
|^deriving from$|[^\\pL\\pM_-]originate from[^\\pL\\pM_-]|^originat
e from[^\\pL\\pM_-]|[^\\pL\\pM_-]originate from$|^originate from$|[
^\\pL\\pM_-]originates from[^\\pL\\pM_-]|^originates from[^\\pL\\pM
_-]|[^\\pL\\pM_-]originates from$|^originates from$|[^\\pL\\pM_-]or
iginated from[^\\pL\\pM_-]|^originated from[^\\pL\\pM_-]|[^\\pL\\pM
_-]originated from$|^originated from$|[^\\pL\\pM_-]originating from
[^\\pL\\pM_-]|^originating from[^\\pL\\pM_-]|[^\\pL\\pM_-]originati
ng from$|^originating from$|[^\\pL\\pM_-]then[^\\pL\\pM_-]|^then[^\
\pL\\pM_-]|[^\\pL\\pM_-]then$|^then$"

that_passive_end_regex="(?<effect>[^:;,!]{2,})(?<connector> that | which |[,]{1}which ){1}(?<ca
use>[^:;,!]{2,})(?<rel_operator>create|creates|creating|created|pro
duce|produces|producing|produced|originate|originates|originating|o
riginated|generate|generates|generated|generating|make|makes|making
|made|destroy|destroys|destroyed|destroying|demolish|demolishes|dem
olished|demolishing|annihilate|annihilates|annihilated|annihilating
|obliterate|obliterates|obliterated|obliterating|cancel|cancels|can
celled|canceled|cancelling|canceling|eradicate|eradicates|eradicate
d|eradicating|extirpate|extirpates|extirpated|extirpating|eliminate
|eliminates|eliminated|eliminating|wipe out|wipes out|wiped out|wip
ing out|wipe off|wipes off|wiped off|wiping off|kill|kills|killed|k
illing|cause|causes|causing|caused|determine|determines|determining
|determined|engender|engenders|engendered|engendering|entail|entail
s|entailed|entailing|provoke|provokes|provoked|provoking|trigger|tr
iggers|triggered|triggering){1}(?<effect_end> in [^:;,!]{2,}| on [^
:;,!]{2,}| since [^:;,!]{2,}| at [^:;,!]{2,}| during [^:;,!]{2,}| t
o [^:;,!]{2,}| for [^:;,!]{2,}| while [^:;,!]{2,}| throughout [^:;,
!]{2,}| all-over [^:;,!]{2,}| all over [^:;,!]{2,}| inside [^:;,!]{
2,}|  outside [^:;,!]{2,}| everywhere [^:;,!]{2,}){0,}[[:punct:]]{0
,}"

creation_verbs_regex_match_all="[^\\pL\\pM_-]create[^\\pL\\pM_
```

```
-]|^create[^\\pL\\pM_-]|[^\\pL\\pM_-]create$|^create$|[^\\pL\\pM_-]
creates[^\\pL\\pM_-]|^creates[^\\pL\\pM_-]|[^\\pL\\pM_-]creates$|^c
reates$|[^\\pL\\pM_-]creating[^\\pL\\pM_-]|^creating[^\\pL\\pM_-]|[
^\\pL\\pM_-]creating$|^creating$|[^\\pL\\pM_-]created[^\\pL\\pM_-]|
^created[^\\pL\\pM_-]|[^\\pL\\pM_-]created$|^created$|[^\\pL\\pM_-]
produce[^\\pL\\pM_-]|^produce[^\\pL\\pM_-]|[^\\pL\\pM_-]produce$|^p
roduce$|[^\\pL\\pM_-]produces[^\\pL\\pM_-]|^produces[^\\pL\\pM_-]|[
^\\pL\\pM_-]produces$|^produces$|[^\\pL\\pM_-]producing[^\\pL\\pM_-
]|^producing[^\\pL\\pM_-]|[^\\pL\\pM_-]producing$|^producing$|[^\\p
L\\pM_-]produced[^\\pL\\pM_-]|^produced[^\\pL\\pM_-]|[^\\pL\\pM_-]p
roduced$|^produced$|[^\\pL\\pM_-]originate[^\\pL\\pM_-]|^originate[
^\\pL\\pM_-]|[^\\pL\\pM_-]originate$|^originate$|[^\\pL\\pM_-]origi
nates[^\\pL\\pM_-]|^originates[^\\pL\\pM_-]|[^\\pL\\pM_-]originates
$|^originates$|[^\\pL\\pM_-]originating[^\\pL\\pM_-]|^originating[^
\\pL\\pM_-]|[^\\pL\\pM_-]originating$|^originating$|[^\\pL\\pM_-]or
iginated[^\\pL\\pM_-]|^originated[^\\pL\\pM_-]|[^\\pL\\pM_-]origina
ted$|^originated$|[^\\pL\\pM_-]generate[^\\pL\\pM_-]|^generate[^\\p
L\\pM_-]|[^\\pL\\pM_-]generate$|^generate$|[^\\pL\\pM_-]generates[^
\\pL\\pM_-]|^generates[^\\pL\\pM_-]|[^\\pL\\pM_-]generates$|^genera
tes$|[^\\pL\\pM_-]generated[^\\pL\\pM_-]|^generated[^\\pL\\pM_-]|[^
\\pL\\pM_-]generated$|^generated$|[^\\pL\\pM_-]generating[^\\pL\\pM
_-]|^generating[^\\pL\\pM_-]|[^\\pL\\pM_-]generating$|^generating$|
[^\\pL\\pM_-]make[^\\pL\\pM_-]|^make[^\\pL\\pM_-]|[^\\pL\\pM_-]make
$|^make$|[^\\pL\\pM_-]makes[^\\pL\\pM_-]|^makes[^\\pL\\pM_-]|[^\\pL
\\pM_-]makes$|^makes$|[^\\pL\\pM_-]making[^\\pL\\pM_-]|^making[^\\p
L\\pM_-]|[^\\pL\\pM_-]making$|^making$|[^\\pL\\pM_-]made[^\\pL\\pM_
-]|^made[^\\pL\\pM_-]|[^\\pL\\pM_-]made$|^made$"

destruction_verbs_regex_match_all="[^\\pL\\pM_-]destroy[^\\pL\\pM_-]|^destroy[^\\pL\
\pM_-]|[^\\pL\\pM_-]destroy$|^destroy$|[^\\pL\\pM_-]destroys[^\\pL\
\pM_-]|^destroys[^\\pL\\pM_-]|[^\\pL\\pM_-]destroys$|^destroys$|[^\
\pL\\pM_-]destroyed[^\\pL\\pM_-]|^destroyed[^\\pL\\pM_-]|[^\\pL\\pM
_-]destroyed$|^destroyed$|[^\\pL\\pM_-]destroying[^\\pL\\pM_-]|^des
troying[^\\pL\\pM_-]|[^\\pL\\pM_-]destroying$|^destroying$|[^\\pL\\
pM_-]demolish[^\\pL\\pM_-]|^demolish[^\\pL\\pM_-]|[^\\pL\\pM_-]demo
lish$|^demolish$|[^\\pL\\pM_-]demolishes[^\\pL\\pM_-]|^demolishes[^
\\pL\\pM_-]|[^\\pL\\pM_-]demolishes$|^demolishes$|[^\\pL\\pM_-]demo
lished[^\\pL\\pM_-]|^demolished[^\\pL\\pM_-]|[^\\pL\\pM_-]demolishe
d$|^demolished$|[^\\pL\\pM_-]demolishing[^\\pL\\pM_-]|^demolishing[
^\\pL\\pM_-]|[^\\pL\\pM_-]demolishing$|^demolishing$|[^\\pL\\pM_-]a
nnihilate[^\\pL\\pM_-]|^annihilate[^\\pL\\pM_-]|[^\\pL\\pM_-]annihi
late$|^annihilate$|[^\\pL\\pM_-]annihilates[^\\pL\\pM_-]|^annihilat
es[^\\pL\\pM_-]|[^\\pL\\pM_-]annihilates$|^annihilates$|[^\\pL\\pM_
-]annihilated[^\\pL\\pM_-]|^annihilated[^\\pL\\pM_-]|[^\\pL\\pM_-]a
nnihilated$|^annihilated$|[^\\pL\\pM_-]annihilating[^\\pL\\pM_-]|^a
nnihilating[^\\pL\\pM_-]|[^\\pL\\pM_-]annihilating$|^annihilating$|
[^\\pL\\pM_-]obliterate[^\\pL\\pM_-]|^obliterate[^\\pL\\pM_-]|[^\\p
L\\pM_-]obliterate$|^obliterate$|[^\\pL\\pM_-]obliterates[^\\pL\\pM
_-]|^obliterates[^\\pL\\pM_-]|[^\\pL\\pM_-]obliterates$|^obliterate
s$|[^\\pL\\pM_-]obliterated[^\\pL\\pM_-]|^obliterated[^\\pL\\pM_-]|
[^\\pL\\pM_-]obliterated$|^obliterated$|[^\\pL\\pM_-]obliterating[^
\\pL\\pM_-]|^obliterating[^\\pL\\pM_-]|[^\\pL\\pM_-]obliterating$|^
obliterating$|[^\\pL\\pM_-]cancel[^\\pL\\pM_-]|^cancel[^\\pL\\pM_-]
|[^\\pL\\pM_-]cancel$|^cancel$|[^\\pL\\pM_-]cancels[^\\pL\\pM_-]|^c
ancels[^\\pL\\pM_-]|[^\\pL\\pM_-]cancels$|^cancels$|[^\\pL\\pM_-]ca
ncelled[^\\pL\\pM_-]|^cancelled[^\\pL\\pM_-]|[^\\pL\\pM_-]cancelled
$|^cancelled$|[^\\pL\\pM_-]canceled[^\\pL\\pM_-]|^canceled[^\\pL\\p
M_-]|[^\\pL\\pM_-]canceled$|^canceled$|[^\\pL\\pM_-]cancelling[^\\p
L\\pM_-]|^cancelling[^\\pL\\pM_-]|[^\\pL\\pM_-]cancelling$|^cancell
ing$|[^\\pL\\pM_-]canceling[^\\pL\\pM_-]|^canceling[^\\pL\\pM_-]|[^
\\pL\\pM_-]canceling$|^canceling$|[^\\pL\\pM_-]eradicate[^\\pL\\pM_
-]|^eradicate[^\\pL\\pM_-]|[^\\pL\\pM_-]eradicate$|^eradicate$|[^\\
```

```
pL\\pM_-]eradicates[^\\pL\\pM_-]|^eradicates[^\\pL\\pM_-]|[^\\pL\\p
M_-]eradicates$|^eradicates$|[^\\pL\\pM_-]eradicated[^\\pL\\pM_-]|^
eradicated[^\\pL\\pM_-]|[^\\pL\\pM_-]eradicated$|^eradicated$|[^\\p
L\\pM_-]eradicating[^\\pL\\pM_-]|^eradicating[^\\pL\\pM_-]|[^\\pL\\
pM_-]eradicating$|^eradicating$|[^\\pL\\pM_-]extirpate[^\\pL\\pM_-]
|^extirpate[^\\pL\\pM_-]|[^\\pL\\pM_-]extirpate$|^extirpate$|[^\\pL
\\pM_-]extirpates[^\\pL\\pM_-]|^extirpates[^\\pL\\pM_-]|[^\\pL\\pM_
-]extirpates$|^extirpates$|[^\\pL\\pM_-]extirpated[^\\pL\\pM_-]|^ex
tirpated[^\\pL\\pM_-]|[^\\pL\\pM_-]extirpated$|^extirpated$|[^\\pL\
\pM_-]extirpating[^\\pL\\pM_-]|^extirpating[^\\pL\\pM_-]|[^\\pL\\pM
_-]extirpating$|^extirpating$|[^\\pL\\pM_-]eliminate[^\\pL\\pM_-]|^
eliminate[^\\pL\\pM_-]|[^\\pL\\pM_-]eliminate$|^eliminate$|[^\\pL\\
pM_-]eliminates[^\\pL\\pM_-]|^eliminates[^\\pL\\pM_-]|[^\\pL\\pM_-]
eliminates$|^eliminates$|[^\\pL\\pM_-]eliminated[^\\pL\\pM_-]|^elim
inated[^\\pL\\pM_-]|[^\\pL\\pM_-]eliminated$|^eliminated$|[^\\pL\\p
M_-]eliminating[^\\pL\\pM_-]|^eliminating[^\\pL\\pM_-]|[^\\pL\\pM_-
]eliminating$|^eliminating$|[^\\pL\\pM_-]wipe out[^\\pL\\pM_-]|^wip
e out[^\\pL\\pM_-]|[^\\pL\\pM_-]wipe out$|^wipe out$|[^\\pL\\pM_-]w
ipes out[^\\pL\\pM_-]|^wipes out[^\\pL\\pM_-]|[^\\pL\\pM_-]wipes ou
t$|^wipes out$|[^\\pL\\pM_-]wiped out[^\\pL\\pM_-]|^wiped out[^\\pL
\\pM_-]|[^\\pL\\pM_-]wiped out$|^wiped out$|[^\\pL\\pM_-]wiping out
[^\\pL\\pM_-]|^wiping out[^\\pL\\pM_-]|[^\\pL\\pM_-]wiping out$|^wi
ping out$|[^\\pL\\pM_-]wipe off[^\\pL\\pM_-]|^wipe off[^\\pL\\pM_-]
|[^\\pL\\pM_-]wipe off$|^wipe off$|[^\\pL\\pM_-]wipes off[^\\pL\\pM
_-]|^wipes off[^\\pL\\pM_-]|[^\\pL\\pM_-]wipes off$|^wipes off$|[^\
\pL\\pM_-]wiped off[^\\pL\\pM_-]|^wiped off[^\\pL\\pM_-]|[^\\pL\\pM
_-]wiped off$|^wiped off$|[^\\pL\\pM_-]wiping off[^\\pL\\pM_-]|^wip
ing off[^\\pL\\pM_-]|[^\\pL\\pM_-]wiping off$|^wiping off$|[^\\pL\\
pM_-]kill[^\\pL\\pM_-]|^kill[^\\pL\\pM_-]|[^\\pL\\pM_-]kill$|^kill$
|[^\\pL\\pM_-]kills[^\\pL\\pM_-]|^kills[^\\pL\\pM_-]|[^\\pL\\pM_-]k
ills$|^kills$|[^\\pL\\pM_-]killed[^\\pL\\pM_-]|^killed[^\\pL\\pM_-]
|[^\\pL\\pM_-]killed$|^killed$|[^\\pL\\pM_-]killing[^\\pL\\pM_-]|^k
illing[^\\pL\\pM_-]|[^\\pL\\pM_-]killing$|^killing$"

cause_verbs_regex_match_all="[^\\pL\\pM_-]cause[^\\pL\\pM_-]|^cause[^\\pL\\pM_-]
|[^\\pL\\pM_-]cause$|^cause$|[^\\pL\\pM_-]causes[^\\pL\\pM_-]|^caus
es[^\\pL\\pM_-]|[^\\pL\\pM_-]causes$|^causes$|[^\\pL\\pM_-]causing[
^\\pL\\pM_-]|^causing[^\\pL\\pM_-]|[^\\pL\\pM_-]causing$|^causing$|
[^\\pL\\pM_-]caused[^\\pL\\pM_-]|^caused[^\\pL\\pM_-]|[^\\pL\\pM_-]
caused$|^caused$|[^\\pL\\pM_-]determine[^\\pL\\pM_-]|^determine[^\\
pL\\pM_-]|[^\\pL\\pM_-]determine$|^determine$|[^\\pL\\pM_-]determin
es[^\\pL\\pM_-]|^determines[^\\pL\\pM_-]|[^\\pL\\pM_-]determines$|^
determines$|[^\\pL\\pM_-]determining[^\\pL\\pM_-]|^determining[^\\p
L\\pM_-]|[^\\pL\\pM_-]determining$|^determining$|[^\\pL\\pM_-]deter
mined[^\\pL\\pM_-]|^determined[^\\pL\\pM_-]|[^\\pL\\pM_-]determined
$|^determined$|[^\\pL\\pM_-]engender[^\\pL\\pM_-]|^engender[^\\pL\\
pM_-]|[^\\pL\\pM_-]engender$|^engender$|[^\\pL\\pM_-]engenders[^\\p
L\\pM_-]|^engenders[^\\pL\\pM_-]|[^\\pL\\pM_-]engenders$|^engenders
$|[^\\pL\\pM_-]engendered[^\\pL\\pM_-]|^engendered[^\\pL\\pM_-]|[^\
\pL\\pM_-]engendered$|^engendered$|[^\\pL\\pM_-]engendering[^\\pL\\
pM_-]|^engendering[^\\pL\\pM_-]|[^\\pL\\pM_-]engendering$|^engender
ing$|[^\\pL\\pM_-]entail[^\\pL\\pM_-]|^entail[^\\pL\\pM_-]|[^\\pL\\
pM_-]entail$|^entail$|[^\\pL\\pM_-]entails[^\\pL\\pM_-]|^entails[^\
\pL\\pM_-]|[^\\pL\\pM_-]entails$|^entails$|[^\\pL\\pM_-]entailed[^\
\pL\\pM_-]|^entailed[^\\pL\\pM_-]|[^\\pL\\pM_-]entailed$|^entailed$
|[^\\pL\\pM_-]entailing[^\\pL\\pM_-]|^entailing[^\\pL\\pM_-]|[^\\pL
\\pM_-]entailing$|^entailing$|[^\\pL\\pM_-]provoke[^\\pL\\pM_-]|^pr
ovoke[^\\pL\\pM_-]|[^\\pL\\pM_-]provoke$|^provoke$|[^\\pL\\pM_-]pro
vokes[^\\pL\\pM_-]|^provokes[^\\pL\\pM_-]|[^\\pL\\pM_-]provokes$|^p
rovokes$|[^\\pL\\pM_-]provoked[^\\pL\\pM_-]|^provoked[^\\pL\\pM_-]|
[^\\pL\\pM_-]provoked$|^provoked$|[^\\pL\\pM_-]provoking[^\\pL\\pM_
-]|^provoking[^\\pL\\pM_-]|[^\\pL\\pM_-]provoking$|^provoking$|[^\\
```

```
pL\\pM_-]trigger[^\\pL\\pM_-]|^trigger[^\\pL\\pM_-]|[^\\pL\\pM_-]tr
igger$|^trigger$|[^\\pL\\pM_-]triggers[^\\pL\\pM_-]|^triggers[^\\pL
\\pM_-]|[^\\pL\\pM_-]triggers$|^triggers$|[^\\pL\\pM_-]triggered[^\
\pL\\pM_-]|^triggered[^\\pL\\pM_-]|[^\\pL\\pM_-]triggered$|^trigger
ed$|[^\\pL\\pM_-]triggering[^\\pL\\pM_-]|^triggering[^\\pL\\pM_-]|[
^\\pL\\pM_-]triggering$|^triggering$|[^\\pL\\pM_-]leads to[^\\pL\\p
M_-]|^leads to[^\\pL\\pM_-]|[^\\pL\\pM_-]leads to$|^leads to$|[^\\p
L\\pM_-]lead to[^\\pL\\pM_-]|^lead to[^\\pL\\pM_-]|[^\\pL\\pM_-]lea
d to$|^lead to$|[^\\pL\\pM_-]led to[^\\pL\\pM_-]|^led to[^\\pL\\pM_
-]|[^\\pL\\pM_-]led to$|^led to$|[^\\pL\\pM_-]leading to[^\\pL\\pM_
-]|^leading to[^\\pL\\pM_-]|[^\\pL\\pM_-]leading to$|^leading to$|[
^\\pL\\pM_-]reason of[^\\pL\\pM_-]|^reason of[^\\pL\\pM_-]|[^\\pL\\
pM_-]reason of$|^reason of$|[^\\pL\\pM_-]result in[^\\pL\\pM_-]|^re
sult in[^\\pL\\pM_-]|[^\\pL\\pM_-]result in$|^result in$|[^\\pL\\pM
_-]results in[^\\pL\\pM_-]|^results in[^\\pL\\pM_-]|[^\\pL\\pM_-]re
sults in$|^results in$|[^\\pL\\pM_-]resulted in[^\\pL\\pM_-]|^resul
ted in[^\\pL\\pM_-]|[^\\pL\\pM_-]resulted in$|^resulted in$|[^\\pL\
\pM_-]resulting in[^\\pL\\pM_-]|^resulting in[^\\pL\\pM_-]|[^\\pL\\
pM_-]resulting in$|^resulting in$|[^\\pL\\pM_-]due to[^\\pL\\pM_-]|
^due to[^\\pL\\pM_-]|[^\\pL\\pM_-]due to$|^due to$|[^\\pL\\pM_-]led
 by[^\\pL\\pM_-]|^led by[^\\pL\\pM_-]|[^\\pL\\pM_-]led by$|^led by$
|[^\\pL\\pM_-]result from[^\\pL\\pM_-]|^result from[^\\pL\\pM_-]|[^
\\pL\\pM_-]result from$|^result from$|[^\\pL\\pM_-]results from[^\\
pL\\pM_-]|^results from[^\\pL\\pM_-]|[^\\pL\\pM_-]results from$|^re
sults from$|[^\\pL\\pM_-]resulted from[^\\pL\\pM_-]|^resulted from[
^\\pL\\pM_-]|[^\\pL\\pM_-]resulted from$|^resulted from$|[^\\pL\\pM
_-]resulting from[^\\pL\\pM_-]|^resulting from[^\\pL\\pM_-]|[^\\pL\
\pM_-]resulting from$|^resulting from$|[^\\pL\\pM_-]stem from[^\\pL
\\pM_-]|^stem from[^\\pL\\pM_-]|[^\\pL\\pM_-]stem from$|^stem from$
|[^\\pL\\pM_-]stems from[^\\pL\\pM_-]|^stems from[^\\pL\\pM_-]|[^\\
pL\\pM_-]stems from$|^stems from$|[^\\pL\\pM_-]stemmed from[^\\pL\\
pM_-]|^stemmed from[^\\pL\\pM_-]|[^\\pL\\pM_-]stemmed from$|^stemme
d from$|[^\\pL\\pM_-]stemming from[^\\pL\\pM_-]|^stemming from[^\\p
L\\pM_-]|[^\\pL\\pM_-]stemming from$|^stemming from$|[^\\pL\\pM_-]d
erive from[^\\pL\\pM_-]|^derive from[^\\pL\\pM_-]|[^\\pL\\pM_-]deri
ve from$|^derive from$|[^\\pL\\pM_-]derives from[^\\pL\\pM_-]|^deri
ves from[^\\pL\\pM_-]|[^\\pL\\pM_-]derives from$|^derives from$|[^\
\pL\\pM_-]derived from[^\\pL\\pM_-]|^derived from[^\\pL\\pM_-]|[^\\
pL\\pM_-]derived from$|^derived from$|[^\\pL\\pM_-]deriving from[^\
\pL\\pM_-]|^deriving from[^\\pL\\pM_-]|[^\\pL\\pM_-]deriving from$|
^deriving from$|[^\\pL\\pM_-]originate from[^\\pL\\pM_-]|^originate
 from[^\\pL\\pM_-]|[^\\pL\\pM_-]originate from$|^originate from$|[^
\\pL\\pM_-]originates from[^\\pL\\pM_-]|^originates from[^\\pL\\pM_
-]|[^\\pL\\pM_-]originates from$|^originates from$|[^\\pL\\pM_-]ori
ginated from[^\\pL\\pM_-]|^originated from[^\\pL\\pM_-]|[^\\pL\\pM_
-]originated from$|^originated from$|[^\\pL\\pM_-]originating from[
^\\pL\\pM_-]|^originating from[^\\pL\\pM_-]|[^\\pL\\pM_-]originatin
g from$|^originating from$"

modals_verbs_regex = "(?<![\\pL\\pM_-])(had ([a-z]{4,} ){1}had to)$|
(?<![\\pL\\pM_-])(do([a-z]{4,} ){1}have to)$|(?<![\\pL\\pM_-])
(does ([a-z]{4,} ){1}have to)$|(?<![\\pL\
\pM_-])(having ([a-z]{4,} ){1}to)$|(?<![\\pL\\pM_-])(did ([a-z]{4,}
 ){1}have to)$|(?<![\\pL\\pM_-])(were ([a-z]{4,} ){1}having to)$|(?
<![\\pL\\pM_-])(will ([a-z]{4,} ){1}have to)$|(?<![\\pL\\pM_-])(hav
e ([a-z]{4,} ){1}had to)$|(?<![\\pL\\pM_-])(has ([a-z]{4,} ){1}had
to)$|(?<![\\pL\\pM_-])(have ([a-z]{4,} ){1}been having to)$|(?<![\\
pL\\pM_-])(had ([a-z]{4,} ){1}been having to)$|(?<![\\pL\\pM_-])(wi
ll ([a-z]{4,} ){1}have had to)$|(?<![\\pL\\pM_-])(would ([a-z]{4,}
){1}have to)$|(?<![\\pL\\pM_-])(would ([a-z]{4,} ){1}have had to)$|
(?<![\\pL\\pM_-])(do ([a-z]{4,} ){1}have to)$|(?<![\\pL\\pM_-])(doe
s ([a-z]{4,} ){1}have to)$|(?<![\\pL\\pM_-])(are ([a-z]{4,} ){1}hav
```

```
ing to)$|(?<![\\pL\\pM_-])(is ([a-z]{4,} ){1}having to)$|(?<![\\pL\
\pM_-])(did ([a-z]{4,} ){1}have to)$|(?<![\\pL\\pM_-])(was ([a-z]{4
,} ){1}having to)$|(?<![\\pL\\pM_-])(were ([a-z]{4,} ){1}having to)
$|(?<![\\pL\\pM_-])(ought ([a-z]{4,} ){1}to)$|(?<![\\pL\\pM_-])(had
 had to)$|(?<![\\pL\\pM_-])(do have to)$|(?<![\\pL\\pM_-])(does hav
e to)$|(?<![\\pL\\pM_-])(having to)$|(?<![\\pL\\pM_-])(did have to)
$|(?<![\\pL\\pM_-])(were having to)$|(?<![\\pL\\pM_-])(will have to
)$|(?<![\\pL\\pM_-])(have had to)$|(?<![\\pL\\pM_-])(has had to)$|(
?<![\\pL\\pM_-])(have been having to)$|(?<![\\pL\\pM_-])(had been h
aving to)$|(?<![\\pL\\pM_-])(will have had to)$|(?<![\\pL\\pM_-])(w
ould have to)$|(?<![\\pL\\pM_-])(would have had to)$|(?<![\\pL\\pM_
-])(do have to)$|(?<![\\pL\\pM_-])(does have to)$|(?<![\\pL\\pM_-])
(are having to)$|(?<![\\pL\\pM_-])(is having to)$|(?<![\\pL\\pM_-])
(did have to)$|(?<![\\pL\\pM_-])(was having to)$|(?<![\\pL\\pM_-])(
were having to)$|(?<![\\pL\\pM_-])(have)$|(?<![\\pL\\pM_-])(had)$|(
?<![\\pL\\pM_-])(has)$|(?<![\\pL\\pM_-])(is)$|(?<![\\pL\\pM_-])(are
)$|(?<![\\pL\\pM_-])(am)$|(?<![\\pL\\pM_-])(was)$|(?<![\\pL\\pM_-])
(were)$|(?<![\\pL\\pM_-])(will)$|(?<![\\pL\\pM_-])(could)$|(?<![\\p
L\\pM_-])(would)$|(?<![\\pL\\pM_-])(should)$|(?<![\\pL\\pM_-])(may)
$|(?<![\\pL\\pM_-])(can)$|(?<![\\pL\\pM_-])(might)$|(?<![\\pL\\pM_-
])(must)$|(?<![\\pL\\pM_-])(shall)$|(?<![\\pL\\pM_-])(ought to)$"


negators_modals_verbs_regex="(?<![\\pL\\pM_-])(had not had to)$|(?
<![\\pL\\pM_-])(do not have to)$|(?<![\\pL\\pM_-])(does not have to
)$|(?<![\\pL\\pM_-])(not having to)$|(?<![\\pL\\pM_-])(did not have
 to)$|(?<![\\pL\\pM_-])(were not having to)$|(?<![\\pL\\pM_-])(will
 not have to)$|(?<![\\pL\\pM_-])(have not had to)$|(?<![\\pL\\pM_-]
)(has not had to)$|(?<![\\pL\\pM_-])(have not been having to)$|(?<!
[\\pL\\pM_-])(had not been having to)$|(?<![\\pL\\pM_-])(will not h
ave had to)$|(?<![\\pL\\pM_-])(would not have to)$|(?<![\\pL\\pM_-]
)(would not have had to)$|(?<![\\pL\\pM_-])(don't have to)$|(?<![\\
pL\\pM_-])(doesn't have to)$|(?<![\\pL\\pM_-])(aren't having to)$|(
?<![\\pL\\pM_-])(isn't having to)$|(?<![\\pL\\pM_-])(didn't have to
)$|(?<![\\pL\\pM_-])(wasn't having to)$|(?<![\\pL\\pM_-])(weren't h
aving to)$|(?<![\\pL\\pM_-])(won't have to)$|(?<![\\pL\\pM_-])(have
n't had to)$|(?<![\\pL\\pM_-])(hasn't had to)$|(?<![\\pL\\pM_-])(ha
ven't been having to)$|(?<![\\pL\\pM_-])(hadn't had to)$|(?<![\\pL\
\pM_-])(hadn't been having to)$|(?<![\\pL\\pM_-])(won't have had to
)$|(?<![\\pL\\pM_-])(wouldn't have to)$|(?<![\\pL\\pM_-])(wouldn't
have had to)$|(?<![\\pL\\pM_-])(shouldn't have to)$|(?<![\\pL\\pM_-
])(shouldn't have had to)$|(?<![\\pL\\pM_-])(should not have to)$|(
?<![\\pL\\pM_-])(should not have had to)$|(?<![\\pL\\pM_-])(may not
 have to)$|(?<![\\pL\\pM_-])(may not have had to)$|(?<![\\pL\\pM_-]
)(not having to)$|(?<![\\pL\\pM_-])(have not)$|(?<![\\pL\\pM_-])(ha
ven't)$|(?<![\\pL\\pM_-])(had not)$|(?<![\\pL\\pM_-])(hadn't)$|(?<!
[\\pL\\pM_-])(hasn't)$|(?<![\\pL\\pM_-])(isn't)$|(?<![\\pL\\pM_-])(
aren't)$|(?<![\\pL\\pM_-])(is not)$|(?<![\\pL\\pM_-])(am not)$|(?<!
[\\pL\\pM_-])(are not)$|(?<![\\pL\\pM_-])(was not)$|(?<![\\pL\\pM_-
])(wasn't)$|(?<![\\pL\\pM_-])(were not)$|(?<![\\pL\\pM_-])(weren't)
$|(?<![\\pL\\pM_-])(will not)$|(?<![\\pL\\pM_-])(won't)$|(?<![\\pL\
\pM_-])(wont)$|(?<![\\pL\\pM_-])(doesn't)$|(?<![\\pL\\pM_-])(don't)
$|(?<![\\pL\\pM_-])(dont)$|(?<![\\pL\\pM_-])(did't)$|(?<![\\pL\\pM_
-])(wouldn't)$|(?<![\\pL\\pM_-])(shan't)$|(?<![\\pL\\pM_-])(shouldn
't)$|(?<![\\pL\\pM_-])(could not)$|(?<![\\pL\\pM_-])(couldn't)$|(?<
![\\pL\\pM_-])(would not)$|(?<![\\pL\\pM_-])(wouldn't)$|(?<![\\pL\\
pM_-])(shouldn't)$|(?<![\\pL\\pM_-])(should not)$|(?<![\\pL\\pM_-])
(may not)$|(?<![\\pL\\pM_-])(can't)$|(?<![\\pL\\pM_-])(cannot)$|(?<
![\\pL\\pM_-])(couldn't)$|(?<![\\pL\\pM_-])(might not)$|(?<![\\pL\\
pM_-])(mustn't)$|(?<![\\pL\\pM_-])(must not)$|(?<![\\pL\\pM_-])(sha
ll not)$|(?<![\\pL\\pM_-])(ought not to)$|(?<![\\pL\\pM_-])(not)$|(
?<![\\pL\\pM_-])(other than)$"
```

```r
#'

RegEx= function(x){
  paste(
    paste("(?<![\\pL\\pM_-])(",
          x,
          ")(?![\\pL\\pM_-])",
          sep = ""),sep="|",collapse = "|"
  )

relation_extractor = function(y,
                        all_at_end_=all_at_end,
                        pattern_ = patterns,
                        passive_pattern_ = passive_pattern,
                        pattern_match_ = pattern_match,
                        pattern_match_all_ = pattern_match_all,
                        that_passive_end_regex_ = that_passive_end_regex,
                        creation_verbs_regex_match_all_= creation_verbs_regex_match_all,
                        destruction_verbs_regex_match_all_=destruction_verbs_regex_match_all,
                        cause_verbs_regex_match_all_=cause_verbs_regex_match_all,
                        modals_verbs_regex_ = modals_verbs_regex,
                        negators_modals_verbs_regex_ =negators_modals_verbs_regex,
                        consider_passive = T,
                        consider_end_form = T) {


  if(is.character(y) && !is.list(y)&& length(y)>1){
    y= quanteda::tokens(y, what = "sentence")
    names(y)=paste(names(y),".",sep = "")
    y= as.list(unlist(quanteda::tokens(y, what = "sentence"),use.names = T,recursive = F))
    names=names(y)
  }

  if(is.character(y) && !is.list(y) && length(y)==1){
    names=names(y)
    y=  as.list(unlist(quanteda::tokens(y, what = "sentence"),use.names = F,recursive = F))
    names(y)= paste(names,".",1:length(y),sep = "")
    names=names(y)
  }

    has_operator  = grepl(pattern_match_,
                         y,
                         perl = T,
                         ignore.case = T)

  operators = list()
  are_if_then = list()
  are_passive = list()
  splits = list()
  removed_if = list()
  negation = list()
  and = list()
  n_relations = list()
  before = list()
  after = list()
  causal_data = list()
  which = list()
  end_form = list()
  contains_end_form=list()
  are_end_form = list()
  contained_end_form = list()
  negated_end_form = list()
```

```
 for (i in which(has_operator)) {

   tryCatch({
     #### match if contains end of sentence form ####
   if(grepl(that_passive_end_regex_,
        y[[i]],
        perl = T,
        ignore.case = T)){
contains_end_form[[i]]=TRUE

#identify and extract end form
end_form[[i]]=gsub(paste("^([^,:!?;]){1,}([,:!?;][[:space:]]){1,}(?=.*( that |
which |[,]{1}which ){1}.*(",all_at_end,"){1}.*)",sep = ""),"",y[[i]],perl = T,
          ignore.case = T)
#end form pruning
end_form[[i]]=   gsub(paste("(.*( that | which |[,]{1}which ){1}.*(",all_at_end,"){1}.*)\\K
([,:!?;][[:space:]]){1,}([^,:!?;]){1,}$",sep = ""),"",end_form[[i]], perl = T,
          ignore.case = T)

#identify different parts of the end form
are_end_form[[i]]  = gregexpr(
                                that_passive_end_regex_,
                                end_form[[i]],
                                perl = T,
                                ignore.case = T
                              )

#extract matches of the end form
   end_form[[i]]  = regcapturedmatches(end_form[[i]],are_end_form[[i]])[[1]]
   if(length(end_form[[i]])>0){
     #paste the effect_end to the effect
   end_form[[i]][,"effect"]= paste(end_form[[i]][,"effect"],end_form[[i]][,"effect_end"],
   sep = "")
   end_form[[i]]  = end_form[[i]][,c("cause","rel_operator","effect")]

   #create relation metadata
   negated_end_form[[i]] =  grepl(
     negators_modals_verbs_regex_,
     end_form[[i]]["effect"],
     ignore.case = T,
     perl = T
   )

   #clean the effect side
   end_form[[i]]["effect"]= gsub(
     negators_modals_verbs_regex_,
     "",
     end_form[[i]]["effect"],
     ignore.case = T,
     perl = T
   )

   #clean the cause side
   end_form[[i]]["cause"]=gsub("[[:space:]]{1,}$",
         "" ,
        end_form[[i]]["cause"],
        ignore.case = T,
        perl = T)
   end_form[[i]]["cause"]=gsub(modals_verbs_regex_,
        "",
        end_form[[i]]["cause"],
```

```
      ignore.case = T,
      perl = T)
  are_end_form[[i]] = data.frame(
    id =  names[i],
    cause = end_form[[i]]["cause"],
    rel_negation = negated_end_form[[i]],
    rel_operator = end_form[[i]]["rel_operator"],
    rel_passive_form = FALSE,
    rel_creation= grepl( creation_verbs_regex_match_all_, end_form[[i]]["rel_operator"],
    ignore.case=T,perl=T ),
    rel_destruction= grepl(destruction_verbs_regex_match_all_, end_form[[i]]["rel_operator"],
    ignore.case=T,perl=T ) ,
    rel_causation =  grepl(cause_verbs_regex_match_all_,end_form[[i]]["rel_operator"],
    ignore.case=T,perl=T ) ,
    rel_coref_res = FALSE,
    effect = end_form[[i]]["effect"],
    #sentence = y[[i]],
    stringsAsFactors  = F,check.names = F,fix.empty.names = F,
    row.names = NULL
  )

 #identify the sentence fragment in which the end_form appears
  end_form[[i]]= base::strsplit(y[[i]],"(?<=[;:,?!.])", perl = TRUE)[[1]]
  contained_end_form[[i]]= !grepl(that_passive_end_regex_,
         end_form[[i]],
         perl = T,
         ignore.case = T)

  if(length(end_form[[i]])>1 && sum(contained_end_form[[i]])>=1){

    #if multiple fragments contained in sentence prune end_form fragment before next processing step
  y[[i]]=  paste(end_form[[i]][contained_end_form[[i]]],sep = "",collapse = "")} else{y[[i]]=""}
  }else{
    contains_end_form[[i]]=FALSE
  }
 }else{
    contains_end_form[[i]]=FALSE
  }

####passive and normal forms ####

  if(grepl(pattern = "[^:;,!]{4,}",y[[i]],perl = T,  ignore.case = T)){

    #extract relation's operators
    operators[[i]]  = regmatches(y[[i]],
                                 gregexpr(
                                   paste(passive_pattern_, pattern_, sep = "|"),
                                   y[[i]],
                                   perl = T,
                                   ignore.case = T
                                 ))[[1]]

    #name the operators NORMAL as default
    names(operators[[i]]) = rep("NORMAL", length(operators[[i]]))

    #identify which ones are passive
    are_passive[[i]] = grepl(
      passive_pattern_,
      unlist(operators[[i]]),
      perl = T,
      ignore.case = T
    )
```

```
  #name these ones PASSIVE
  names(operators[[i]])[are_passive[[i]]] = "PASSIVE"

  #if there is at least one passive process string as follows
  if (length(operators[[i]][are_passive[[i]]]) > 0) {
    splits[[i]] = regmatches(
      y[[i]],
      gregexpr(
        RegEx(c(operators[[i]][are_passive[[i]]], operators[[i]][!are_passive[[i]]])),
        y[[i]],
        perl = T,
        ignore.case = T
      ),
      invert = T
    )[[1]]

  } else{ #if no passive process string as follows
    splits[[i]] = regmatches(
      y[[i]],
      gregexpr(
        RegEx(operators[[i]]),
        y[[i]],
        perl = T,
        ignore.case = T
      ),
      invert = T
    )[[1]]
  }



#####BUILD PAIRS ####
if (length(operators[[i]]) > 0) {
  splits[[i]] = gsub("^([[:punct:][:space:]]){1,}|([[:punct:][:space:]]){1,}$",
                     "",
                     splits[[i]])
  negation[[i]] = grepl(
    negators_modals_verbs_regex_,
    splits[[i]],
    ignore.case = T,
    perl = T
  )
  splits[[i]] = gsub(
    negators_modals_verbs_regex_,
    "",
    splits[[i]],
    ignore.case = T,
    perl = T
  )
  splits[[i]] = gsub(modals_verbs_regex_,
                     "",
                     splits[[i]],
                     ignore.case = T,
                     perl = T)
  and[[i]] = grepl(and_regex,
                   splits[[i]],
                   perl = T,
                   ignore.case = T)
  splits[[i]] = gsub(
    and_regex,
    "",
```

```
    splits[[i]],
    perl = T,
    ignore.case = T
  )

  which[[i]] = grepl("which[[:space:]]{0,}$",
                     splits[[i]],
                     ignore.case = T,
                     perl = T)
  splits[[i]] = gsub(
    "which[[:space:]]{0,}$|that[[:space:]]{0,}$",
    "",
    splits[[i]],
    perl = T,
    ignore.case = T
  )
  splits[[i]] = gsub(
    "^[[:space:][:punct:]]{0,}|[[:space:][:punct:]]{0,}$",
    "",
    splits[[i]],
    perl = T,
    ignore.case = T
  )
  before[[i]] = splits[[i]][1:length(operators[[i]])]
  after[[i]] = splits[[i]][2:(length(operators[[i]]) + 1)]
  n_relations[[i]] = length(splits[[i]]) - 1
  for (j in  1:n_relations[[i]]) {
    k = j
    if (!are_passive[[i]][j]) {
      if (j == 2 && (and[[i]][j] | which[[i]][j - 1])) {
        k = 1
        before[[i]][j] = splits[[i]][k]
        after[[i]][j] = splits[[i]][j + 1]
      } else{
        if (j > 2 && (and[[i]][j] | which[[i]][j - 1])) {
          k = max(which(!and[[i]][1:j - 1] & !which[[i]][1:j - 1]))
          before[[i]][j] = splits[[i]][k]
          after[[i]][j] = splits[[i]][j + 1]
        }
      }
    } else{
      if (j == 2 && (and[[i]][j] | which[[i]][j - 1])) {
        k = 1
        after[[i]][j] = splits[[i]][k]
        before[[i]][j] = splits[[i]][j + 1]
      } else{
        if (j > 2 && (which[[i]][j - 1])) {
          k = max(which(!which[[i]][1:j - 1]))
          after[[i]][j] = splits[[i]][k]
          before[[i]][j] = splits[[i]][j + 1]
        } else{
          after[[i]][j] = splits[[i]][k]
          before[[i]][j] = splits[[i]][k + 1]
        }
      }
    }
  }

  causal_data[[i]] = data.frame(
    id =  names[i],
    cause = before[[i]][1:n_relations[[i]]],
    rel_negation = negation[[i]][1:n_relations[[i]]],
```

```
        rel_operator = operators[[i]][1:n_relations[[i]]],
        rel_passive_form = are_passive[[i]][1:n_relations[[i]]],
        rel_creation= grepl( creation_verbs_regex_match_all_,
        operators[[i]][1:n_relations[[i]]], ignore.case=T,perl=T ),
        rel_destruction= grepl(destruction_verbs_regex_match_all_,
        operators[[i]][1:n_relations[[i]]], ignore.case=T,perl=T ) ,
        rel_causation =  grepl(cause_verbs_regex_match_all_,operators[[i]][1:n_relations[[i]]],
        ignore.case=T,perl=T ) ,
        rel_coref_res = and[[i]][1:n_relations[[i]]] | which[[i]][1:n_relations[[i]]],
        effect = after[[i]][1:n_relations[[i]]],
        #sentence = y[[i]],
        stringsAsFactors  = F,check.names = F,fix.empty.names = F,row.names = NULL
      )
      if(contains_end_form[[i]]){
        causal_data[[i]]= rbind(are_end_form[[i]],causal_data[[i]])
    }

    }else{
      if(contains_end_form[[i]]){
        causal_data[[i]]= are_end_form[[i]]
      }
    }
    }else{
      if(contains_end_form[[i]]){
        causal_data[[i]]= are_end_form[[i]]
      }
    }

    })
  }
  dplyr::bind_rows(causal_data)
}
```
