# Supplementary material for: The architecture of partisan debates: The online controversy on the no-deal Brexit
Source: PLoS One. 2022 Jun 30;17(6):e0270236. doi: 10.1371/journal.pone.0270236 (PMC9246126; doi:10.1371/journal.pone.0270236)
Supplement: Attachment — Submitted filename: Response to Reviewers.pdf [file pone.0270236.s002.pdf]

Reply to the Academic Editor and reviewers

We sincerely thank the Academic Editor and the reviewers for carefully reading our paper and providing very helpful comments. Our responses to these comments and suggestions are preceded by the symbol '□'; the editor's and reviewers' comments appear in **bold**.

Reply to Academic Editor

* **In agreement with the reviewers' comments I recommend to focus a particular attention on contextualization of the paper in the state of the art, on figures' quality and on a more careful explanation of the methodologies. For this last point please keep in mind the interdisciplinary scope of the journal and make sure that the methodologies are well explained for a public potentially larger than for a disciplinary journal.**

□ *To better contextualize the paper in the state of the art, we have enriched the Introduction section by adding references and by improving the description of the methodological background of the paper and its limits. Please refer to the reply to comments 5 and 6 by reviewer 2.*

For what concerns the quality of figure previews, please refer to the reply to comment 1 by reviewer 1.

Finally, we have reviewed the methodological section, adding simplified explanations and references to introductory readings, to make it more accessible to a large scientific audience.

Reply to reviewer 1 (Manoel Horta Ribeiro)

1. **I have found the quality of images to be below the acceptable standards for scientific publication. Figures 1 *(probably refers to Figure 3)* and 2 should have legends explaining the meaning on the line in the Figure (and not only in the caption). Figures and tables in general should be .pdfs or high quality images.**

□ *We thank the reviewer for the remark. We are sorry for the low quality of the Figure previews provided at the end of the submitted article. Unfortunately, we have no control on the resolution and blurring of these images. To see the original .tif Figures that were uploaded during the submission on the portal of PLOS ONE, please use the links on the top right corner of each Figure preview at the end of the article. As the reviewer correctly pointed out, these (resized) preview images shown at the end of the article have a non satisfactory appearance and wouldn't be appropriate for publication. But, this is an artifact of the submission processing system which, we believe, should not impact the quality of the images in the final publication, which are the ones accessible through the links. The fact that the preview images look so blurry is probably due to the fact that they were not natively in vector graphics, because the R libraries used to plot networks (visnetwork) and tables don't allow to export them in vector graphics formats (see GitHub discussion: https://github.com/datastorm-open/visNetwork/issues/204), which are required by PLOS ONE. Therefore, we had to convert all Figures in a supported format (.tif) using an image editing software. As a result, when .tif Figures are uploaded on the PLOS ONE portal, and automatically resized by the submission processing system, their previews look blurred even if uploaded images meet the required resolution and format standards. To further improve the quality of some Figures (e.g., those containing multiple sub-plots of networks) we have recreated and exported them at a higher resolution, and checked again on PACE that all figures meet the PLOS ONE*

requirements checklist. For what concerns Figures 2 and 3, following the suggestions of the reviewer, captions and legends now explain the meaning of all the lines:

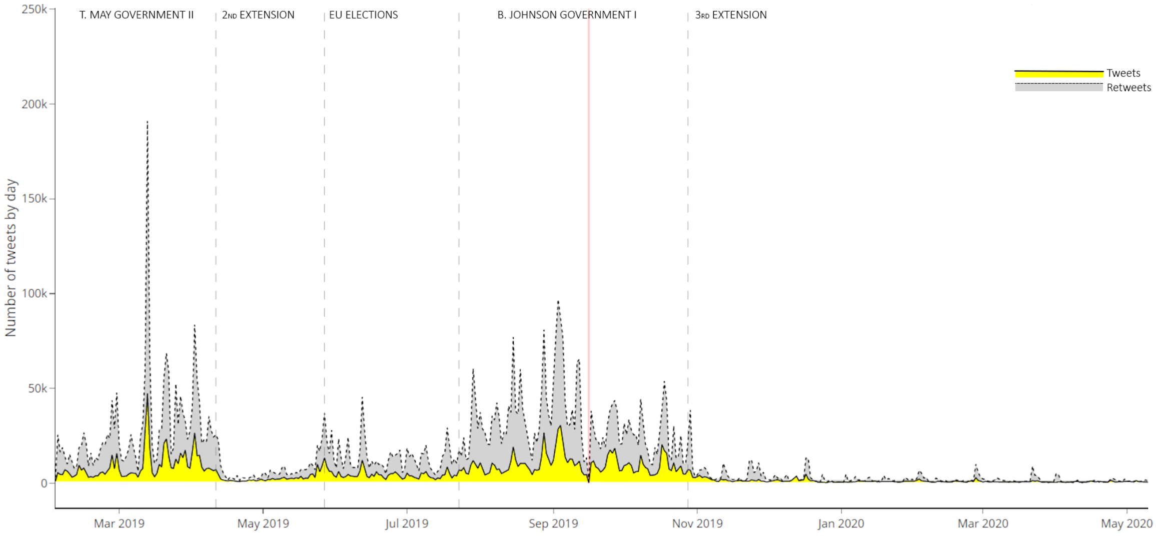

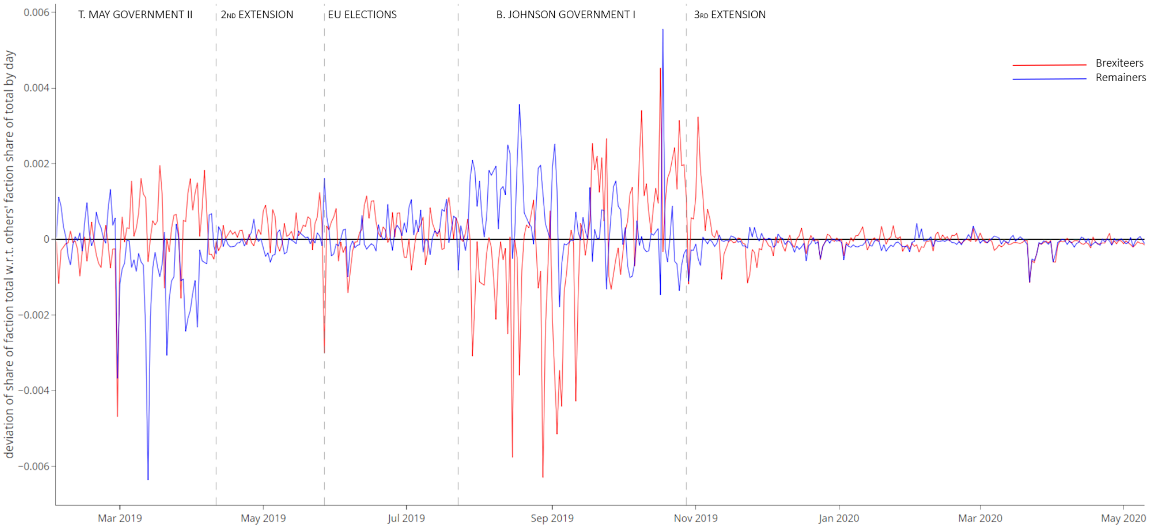

2. **I appreciated the usage of STMs and the methodology to create the topic network in Figure 5. But the effect sizes on the differences captured by the edge colors are unclear to me. Authors should indicate the effect size either in the figure (at least for the discussed edges) or in the text (as they**

discuss bigrams).

☐ *We thank you for the comment and we are glad that the reviewer has appreciated the usage of STM for identifying topic specific differences in probabilities of words and bigrams by faction. In Figure 5 we used a red-gray-blue color scale to highlight node (term) and edge (bigram) differences, in terms of topic and faction specific probabilities, between Brexiteers and Remainers. The scale uses gray for values close to 0 and has as positive and negative extremum the absolute value of the highest observed difference among nodes/edges between the two partisan factions (extremum values are shown in the legend).*

Since indicating the effect size (for each word and bigram) in Figure 5 would make it too difficult to read, in order to facilitate the interpretation of the Figure and related results we have provided in the text of the article the effect size of the difference for the discussed bigrams.

At the reviewer's best convenience, we here report the excerpts from edited paragraphs in the Results section:

> *The terms "shortages", "food" and "medicine" are among the most relevant terms for non-partisan users (see box n.1 in Figure 5). The probabilities of these terms and collocations are significantly different for Brexiteers and Remainers. For example, while Brexiteers focus more on the term "food" (*0.1272 *vs.* 0.0438*) and the collocation "food → shortages" (*0.0645 *vs.* 0.0398*) , Remainers focus relatively more on the terms "medicine" (*0.0411 *vs.* 0.0038*) and "medication" (*0.0202 *vs.* 0.0019*) and the collocations "medicine → shortages" (*0.0307 *vs.* 0.0023*) and "medication → shortages" (*0.0203 *vs.* 0.0019*) .*
>
> *The Remainers also tend to focus more on the possible effects on unemployment of a no-deal scenario (see box n.2); both the unigrams "job" (*0.0566 *vs.* 0.0141*) and "losses" (*0.0283 *vs.* 0.0116*) and the bigram "job → losses" (*0.0289 *vs.* 0.0126*) have higher probabilities for Remainers than Brexiteers.*

*Finally, box n. 4 shows that members of partisan factions are more inclined to talk about the party representing the other faction with respect to their own: Remainers use relatively more frequently the terms "tory" (*0.00048 *vs.* 0.00026*) and "tory → party" (*0.00013 *vs.* 0.00009*) , whereas Brexiteers use more frequently the terms "labour" (*0.00066 *vs.* 0.00011*) and "labour → party" (*0.00008 *vs.* 0.00007*). This signals that while debating about the effects of no-deal scenario, partisan faction members also argue about the no-deal narratives employed by the opposing faction. The aforementioned results highlight that the proposed methodology can capture topic-specific argumentation differences between factions also at the phrasal (and phrase fragment) level.*

3. **In Figure 6 I found the way in which you explained the topic ordering on the y-axis confusing. In my view it would make things clearer if you clarified that these correlations can be obtained in a STM by construction.**

□ *We thank the reviewer for the remark. We agree that the STM allows for estimating correlations among topics. However, the the decomposability of the correlation matrix in blocks that reveal the presence of polarised (Destruction and Creation) verb-group assortativity among topics is not obtained by construction, and we consider this result a major finding. The topic ordering on both axes (x and y) is based on the topic-specific difference between the values of the polar verb-type coefficients (Destruction vs. Creation), inferred with STM. Topics with non significant differences in the polar verb-group coefficients are in yellow. Topics whose probability of being observed is significantly higher conditional on having a Creation verb are in green. Topics whose probability of being observed is significantly higher conditional on having a Destruction verb are in red. Coefficient values are in brackets (only for statistically significant differences). The reason for using a redundant ordering in the two axes is to visualize the decomposability of the matrix in blocks that reveal the presence of polar verb-type*

assortativity. To make the explanation of the topic ordering in Figure 6 clearer, we have expanded the explanation of this Figure in the text of the article. At the reviewer's best convenience, we here report the excerpts from added paragraphs in the Results section:

> *The topic ordering on both axes of Figure Figure 6 is based on topic-specific differences between the coefficients of the two polarised verb groups (Destruction vs. Creation), inferred with STM. Such ordering allows us to appraise to which degree arguments that are characterised by the same polarised verb group are more likely to occur together in causal tweets related to the no-deal, with respect to pairs of arguments that are characterized by different verb groups.*

4. **I didn't get what additional insights came from Figure 7. This in my opinion is a weak result that did not add much to the paper. I get that you show there is assortativity between topics (which is already shown in 6) and between groups (i.e, Brexiteers vs. Remainers, which is new) —but wouldn't it be much easier to simply re-do Figure 6 for the groups?**

☐ *We thank the reviewer for the remark. The reason to provide Figure 7 is to highlight through a single image the building blocks emerging from different levels of filtration (Chazal and Michel, 2021) and to display the topic (typing) dimensions not captured by figure 6 (e.g., factions and negations) and the structural balance of topic triads (Cartwright and Harary, 1956). Therefore, redoing Figure 6 for the groups (i.e., factions) would certainly help evaluate the correlation between topics characterising brexiteers and remainers, but would not allow us to identify multi-dimensional assortative relations between topics, as desired.*

Reply to reviewer 2 (anonymous reviewer)

5. **Concerning the need to better insert the work in the reference literature, I invite the authors to enrich the bibliography and to justify many of the statements present along the work, especially in the introduction, which at the moment do not find support in the bibliography.**

   - **line 22-24: 'Public debates [. . .] are strategic discussions about socially relevant issues.' Did somebody use this definition before? Is it always the case? Are we always strategic when we write online? I wouldn't say so.**
   - **line 35-36: 'These can be related...them'. There are many works on the endogenous and exogenous effects on online attention dynamics.**
   - **line 93: 'the no-deal aspect has polarised public opinions and received great media attention'. Many works have been written about polarization during the Brexit debate.**
   - **line 47: 'The proposed framework builds on existing litterature'. I think that it would be better to clearly state the previous works here, in order to give the expert reader some clear references. So instead of citing [22], [23], [24] in the Method section for the first time, I would suggest to anticipate them in the introduction to give a clearer context to the reader.**
   - **line 94-96: even though it's clear the no-deal debate played a crucial role in the 2019 UK elections, citing some literature would make the statement stronger.**

☐ *We thank the reviewer for his remarks and reference suggestions. Following his comments we have enriched the references in the Introduction section of the article, as well as the explanations of related works in the literature, this to better illustrate the*

article's research background and justify the statements identified by the reviewer.
For what concerns the reviewer's remark on the strategic dimension of debates. By 'strategic' we do not mean that all individuals necessarily behave rationally, but that the success of their argumentation choices will likely depend on the strategic interaction and interdependence with the arguments employed by others, this above the fact that individuals will decide which arguments to use rationally or in a emotionally driven way. We added a paragraph in the Introduction of the article to explain this point. We thank the reviewer for his questions, which pushed us to clarify this aspect in the article. At the reviewer's best convenience, we here report the excerpts from added/edited paragraphs in the Introduction section:

> *At the individual level, debaters don't necessarily behave rationally but they are part of a strategic interaction. For example, they don't necessarily take into account all others' argumentation strategies, political preferences and incentives when choosing which arguments to use. However, the outcomes of their argumentation choices are interdependent from the substantial point of view, since their success (e.g., diffusion and consent) and their effects on third party opinions are generally interdependent. Therefore, interactions among debaters' argumentation choices are strategic from a substantial perspective (Goffman, 1970). This is true even when single arguments are seemingly chosen intuitively, in an emotionally driven way or even randomly.*
> *Arguments can be used for a variety of purposes, like persuading an audience, orienting opinions or justifying decisions, both ex-ante or ex-post. Issues in debates always incorporate a controversial component (Garimella et al., 2018), associated with partisan factions that support specific views or resolutions (Adamic and Glance, 2005). [...]*
>
> *These can be related both to the endogenous dynamics of the public debate (Crane and Sornette, 2008; Leskovec et al., 2009), such as policy*

communications by elected officials (Dybowski and Adämmer, 2018), and to external events that potentially steer them, like extreme natural events (Kühne et al., 2021) or terrorist attacks (Fischer-Preßler et al., 2019). [...]

The proposed framework builds on existing literature(Blei and Lafferty, 2006; Mimno and McCallum, 2008; Eisenstein et al., 2011) [...]

The online debate on Brexit is an appealing case to investigate with the proposed methodology, because, since the 2016 referendum, the "no-deal" aspect has polarised public opinions and received great media attention (Del Vicario et al., 2017; Hobolt et al., 2021), both offline and online. Besides, until the last extension granted by the EU Council (28 October 2019), which preceded the 2019 general elections in the UK, citizens' attitudes towards a no-deal scenario were a polarising dimension of the Brexit debate(Cooper and Cooper, 2020; Toszek, 2020; Flinders, 2020).

6. **Concerning the discussion of the limitations I would suggest to avoid some unjustified generalizations and I would like the authors to better discuss the limitations of their work.Two points should be discussed in my opinion:**

   (a) **The limit of studying the public debate on Twitter. Studying Twitter does not imply studying society. I would like the authors to stress in the discussion that analyzing the public debate on twitter could be profoundly different from studying the public debate on the no-deal Brexit in general.**

   (b) **I would avoid the first sentence of the Discussion: "The present work shows that the structure and dynamics of online debates connect arguments in a coherent way across different levels and roles". Is the online debate always rational and coherent? The authors identified 361116 relations on over 9 million Tweets. It seems that the majority**

of the tweets does not contain causal argumentations... I would like to read more about possible interpretations of why only few tweets have structured argumentations.

☐ *We thank the reviewer for comment 2.(a). To answer this point, we have expanded the literature review, highlighting the limits of studying the public debate on Twitter, in particular with respect to representativeness biases. At the reviewer's best convenience, we here report the excerpts from added/edited paragraphs in the Introduction section:*

> *It has been found that social media communities constitute a non-representative sample of the population (Blank and Lutz, 2017) and exhibit biases in terms of age, gender, and education (Mellon and Prosser, 2017). Being predominantly made by highly educated and sociopolitically active individuals, social media ecosystems like the Twittersphere (Rogers, 2013) reveal a wide and non-uniform spectrum of public opinions on issues of collective interest and concern (Ausserhofer and Maireder, 2013). While aware of the problems of generalizability and limits of Web data based research (Tufekci, 2014; Caldarelli et al., 2014; Gayo-Avello, 2012), we believe that online debates on social media platforms, being fully observable, offer a privileged window on the argumentation dynamics of a relatively large and varied sample of the politically engaged population (Sudhahar et al., 2015), which comprises key influencers of the public sphere.*
> *[...]*

☐ *We thank the reviewer for comment 2.(b). Our main goal was not to display and analyse a variety of arguments, but to focus on the analysis of a specific -and in our opinion very relevant- type of argumentation, that is causal arguments. We have made this point clearer in the Introduction of the article. The number of arguments that we identify is therefore voluntarily limited and doesn't consider, for example, modal verbs*

and other arguments related to influence, facilitation or obstruction. However, the proposed Regex based argument extraction method, which is now available as an open source OpenAPI (`https://github.com/carlosantagiustina/ISEED-AEA`), can be adapted to capture additional argumentation structures with relative little effort. However, we believe that considering all types of arguments together in this paper would have probably blurred the results and rendered the article longer and more complicated to understand.

As shown in Figure 2, large share of the over 9 million tweets were retweets, that we voluntary omit in the arguments analysis. This to avoid that replications of existing posts could heavily influence the STM results. Moreover, a large share of tweets likely contain emotional reactions to Brexit related news, factual statements assertions or value judgements related to the no-deal debate. Extending the analysis to those dimensions of this debate would certainly be interesting but goes beyond the aims and scope of the current contribution. However, we will certainly consider it in future works and we thank the reviewer for this suggestion. Also, the fact that there are statistically significant differences in the relative frequency of the usage of causal arguments among the two partisan factions is an important finding, which suggests the usage of causal arguments may be related to some user/group characteristics, hypothetically including political alignment, among other demographic and socio-cultural characteristics.

For what concerns the identification of the emotional aspect of the Tweets, it is not among the objectives of this work. While extending our analysis to emotional aspects could be possible - and already some topics are characterised by emotional words and emojis - an emotion and sentiment analysis would go beyond the scope and methodological contribution of this article.

Finally, to first sentence of the Discussion has been edited to make it more focused and

adherent to the proposed methodology and related findings, we thank the reviewer for his suggestion.
At the reviewer's best convenience, we here report the excerpts from added/edited paragraphs in the Introduction and Discussion sections:

> *[Introduction] The focus was placed on causal relations as a prominent example of argumentation. Although they represent a limited and very specific aspect of debates, these arguments are key for understanding (elicited) causal representations of online groups. In particular, in relation to issues of collective interest having to do with hypothetical (extreme) scenarios related to the failure in reaching an agreement among negotiating parties with conflicting interests, such as during the Brexit or the 2022 war between Ukraine and Russia. However, the proposed approach can be extended to other types of arguments, such as permission, facilitation, obstruction, possibility and influence, which are widely employed in debates (Talmy, 1988).*
> *[...]*
>
> *The present work shows that the structure and dynamics of the debate bind arguments in a multi-level "architecture". This architecture integrates the analysis of interdependent arguments, their factional specificities, and leading/following roles of partisan factions. Moreover, the current reconstruction of causal arguments in the no-deal debate allows to unveil how the debate dynamics relate to external events and how they are shaped by factional interests. For example, Remainers resort more extensively to causal arguments than Brexiteers do, they emphasise the potential destructive causal effects of the no-deal Brexit, and stress its (negative) economic implications.*

References

Adamic, L. A. and Glance, N. (2005). The political blogosphere and the 2004 us election: divided they blog. In *Proceedings of the 3rd international workshop on Link discovery*, pages 36–43.

Ausserhofer, J. and Maireder, A. (2013). National politics on twitter: Structures and topics of a networked public sphere. *Information, communication & society*, 16(3):291–314.

Blank, G. and Lutz, C. (2017). Representativeness of social media in great britain: investigating facebook, linkedin, twitter, pinterest, google+, and instagram. *American Behavioral Scientist*, 61(7):741–756.

Blei, D. and Lafferty, J. (2006). Correlated topic models. *Advances in neural information processing systems*, 18:147.

Caldarelli, G., Chessa, A., Pammolli, F., Pompa, G., Puliga, M., Riccaboni, M., and Riotta, G. (2014). A multi-level geographical study of italian political elections from twitter data. *PloS one*, 9(5):e95809.

Cartwright, D. and Harary, F. (1956). Structural balance: a generalization of heider's theory. *Psychological review*, 63(5):277.

Chazal, F. and Michel, B. (2021). An introduction to topological data analysis: fundamental and practical aspects for data scientists. *Frontiers in Artificial Intelligence*, 4.

Cooper, L. and Cooper, C. (2020). 'get brexit done': The new political divides of england and wales at the 2019 election. *The Political Quarterly*, 91(4):751–761.

Crane, R. and Sornette, D. (2008). Robust dynamic classes revealed by measuring the response function of a social system. *Proceedings of the National Academy of Sciences*, 105(41):15649–15653.

Del Vicario, M., Zollo, F., Caldarelli, G., Scala, A., and Quattrociocchi, W. (2017). Mapping social dynamics on facebook: The brexit debate. *Social Networks*, 50:6–16.

Dybowski, T. and Adämmer, P. (2018). The economic effects of us presidential tax communication: Evidence from a correlated topic model. *European Journal of Political Economy*, 55:511–525.

Eisenstein, J., Ahmed, A., and Xing, E. P. (2011). Sparse additive generative models of text. In *Proceedings of the 28th international conference on machine learning (ICML-11)*, pages 1041–1048. Citeseer.

Fischer-Preßler, D., Schwemmer, C., and Fischbach, K. (2019). Collective sense-making in times of crisis: Connecting terror management theory with twitter user reactions to the berlin terrorist attack. *Computers in Human Behavior*, 100:138–151.

Flinders, M. (2020). Not a brexit election? pessimism, promises and populism 'uk-style'. *Parliamentary Affairs*, 73(Supplement_1):225–242.

Garimella, K., Morales, G. D. F., Gionis, A., and Mathioudakis, M. (2018). Quantifying controversy on social media. *ACM Transactions on Social Computing*, 1(1):1–27.

Gayo-Avello, D. (2012). No, you cannot predict elections with twitter. *IEEE Internet Computing*, 16(6):91–94.

Goffman, E. (1970). *Strategic interaction*, volume 1. University of Pennsylvania Press.

Hobolt, S. B., Leeper, T. J., and Tilley, J. (2021). Divided by the vote: Affective polarization in the wake of the brexit referendum. *British Journal of Political Science*, 51(4):1476–1493.

Kühne, O., Koegst, L., Zimmer, M.-L., and Schäffauer, G. (2021). "... inconceivable, unrealistic and inhumane". internet communication on the flood disaster in west germany of july 2021 between conspiracy theories and moralization—a neopragmatic explorative study. *Sustainability*, 13(20):11427.

Leskovec, J., Backstrom, L., and Kleinberg, J. (2009). Meme-tracking and the dynamics of the news cycle. In *Proceedings of the 15th ACM SIGKDD international conference on Knowledge discovery and data mining*, pages 497–506.

Mellon, J. and Prosser, C. (2017). Twitter and facebook are not representative of the general population: Political attitudes and demographics of british social media users. *Research & Politics*, 4(3):2053168017720008.

Mimno, D. M. and McCallum, A. (2008). Topic models conditioned on arbitrary features with dirichlet-multinomial regression. In *UAI*, volume 24, pages 411–418. Citeseer.

Rogers, R. (2013). Debanalizing twitter: The transformation of an object of study. In *Proceedings of the 5th annual ACM web science conference*, pages 356–365.

Sudhahar, S., Veltri, G. A., and Cristianini, N. (2015). Automated analysis of the us presidential elections using big data and network analysis. *Big Data & Society*, 2(1):2053951715572916.

Talmy, L. (1988). Force dynamics in language and cognition. *Cognitive Science*, 12(1):49–100.

Toszek, B. H. (2020). The battle of brexit. analysis of the 2019 united kingdom general election results. *Polish Political Science Yearbook*, 49(4):153–166.

Tufekci, Z. (2014). Big questions for social media big data: Representativeness, validity and other methodological pitfalls. In *Eighth international AAAI conference on weblogs and social media*, pages 505–514.
